# *RUNX1* mutations promote leukemogenesis of myeloid malignancies in *ASXL1*-mutated leukemia

**DOI:** 10.1186/s13045-019-0789-3

**Published:** 2019-10-22

**Authors:** Rabindranath Bera, Ming-Chun Chiu, Ying-Jung Huang, Tung-Huei Lin, Ming-Chung Kuo, Lee-Yung Shih

**Affiliations:** 1 Division of Hematology-Oncology, Chang Gung Memorial Hospital at Linkou, Taoyuan, Taiwan; 2 College of Medicine, Chang Gung University, Taoyuan, Taiwan; 3 Division of Hematology-Oncology, Chang Gung Memorial Hospital, 199, Tung-Hwa North Road, Taipei, Taiwan 10590

**Keywords:** ASXL1, RUNX1, Myeloid transformation, HIF-1α, ID1

## Abstract

**Background:**

Additional sex combs-like 1 (*ASXL1*) mutations have been described in all forms of myeloid neoplasms including chronic myelomonocytic leukemia (CMML) and associated with inferior outcomes, yet the molecular pathogenesis of *ASXL1* mutations (*ASXL1*-MT) remains poorly understood. Transformation of CMML to secondary AML (sAML) is one of the leading causes of death in CMML patients. Previously, we observed that transcription factor *RUNX1* mutations (*RUNX1*-MT) coexisted with *ASXL1*-MT in CMML and at myeloid blast phase of chronic myeloid leukemia. The contribution of *RUNX1* mutations in the pathogenesis of myeloid transformation in *ASXL1*-mutated leukemia, however, remains unclear.

**Methods:**

To evaluate the leukemogenic role of RUNX1-MT in *ASXL1*-mutated cells, we co-expressed *RUNX1*-MT (R135T) and *ASXL1*-MT (R693X) in different cell lines and performed immunoblot, co-immunoprecipitation, gene expression microarray, quantitative RT-PCR, cell proliferation, differentiation, and clonogenic assays for in vitro functional analyses. The in vivo effect was investigated using the C57BL/6 mouse bone marrow transplantation (BMT) model.

**Results:**

Co-expression of two mutant genes increased myeloid stem cells in animal model, suggesting that cooperation of *RUNX1* and *ASXL1* mutations played a critical role in leukemia transformation. The expression of *RUNX1* mutant in *ASXL1*-mutated myeloid cells augmented proliferation, blocked differentiation, and increased self-renewal activity. At 9 months post-BMT, mice harboring combined *RUNX1* and *ASXL1* mutations developed disease characterized by marked splenomegaly, hepatomegaly, and leukocytosis with a shorter latency. Mice transduced with both *ASXL1* and *RUNX1* mutations enhanced inhibitor of DNA binding 1 (ID1) expression in the spleen, liver, and bone marrow cells. Bone marrow samples from CMML showed that *ID1* overexpressed in coexisted mutations of *RUNX1* and *ASXL1* compared to normal control and either *RUNX1*-MT or *ASXL1*-MT samples. Moreover, the RUNX1 mutant protein was more stable than WT and increased HIF1-α and its target *ID1* gene expression in *ASXL1* mutant cells.

**Conclusion:**

The present study demonstrated the biological and functional evidence for the critical role of *RUNX1*-MT in *ASXL1*-mutated leukemia in the pathogenesis of myeloid malignancies.

## Background

Somatic mutations of additional sex comb-like protein 1 (*ASXL1*) gene have been described in patients with various types of myeloid malignancies, including myelodysplastic syndromes (MDS) (15–25%), myeloproliferative neoplasms (MPN) (10–15%), 40% of chronic myelomonocytic leukemia (CMML), a few patients with chronic myeloid leukemia (CML), and 15–20% of acute myeloid leukemia (AML) [1–5]. *ASXL1* mutations also have been detected in myeloid blast crisis (BC) of CML patients [5]. CMML is a clonal hematological disorder characterized by monocytosis, dysplasia, and an increased risk of progression to secondary acute myeloid leukemia (sAML) [6, 7]. Transformation of CMML to sAML is one of the leading causes of death in CMML patients and has been associated with genetic alterations that may contribute to the leukemic transformation of CMML [8, 9]. However, the molecular pathogenesis of the progression of CMML to sAML remains unclear.

CMML has been associated with somatic mutations in various identified genes involving epigenetic regulators, spliceosome components, transcription factors (RUNX1), and cell signaling [6, 8, 9]. Among these, C-terminal-truncating *ASXL1* mutations (frameshift and nonsense) were associated with inferior overall survival and a high risk of AML transformation in MDS and CMML [1, 2, 4, 10, 11]. Previous data demonstrated that ASXL1 interacts with components of the polycomb complex PRC2, namely EZH2 and SUZ12, and inhibition of ASXL1 function leading to loss of H3K27me3 histone marks [2]. In addition to H3K27me3, recent studies have shown that ASXL1 is involved in the regulation of H2AK119 ubiquitination through interactions with BAP1 and/or BMI1 [12, 13]. Moreover, previous data using the murine model have shown that C-terminal-truncating ASXL1 mutants inhibit myeloid differentiation and induce an MDS-like disease [14]. Recently, Yang et al. reported that truncated ASXL1 protein functions as a gain-of-function to promote the pathogenesis of myeloid malignancies using the transgenic mouse model [15]. We have previously found a high frequency of *RUNX1* mutations in CMML patients [16]. We also observed that *RUNX1* and *ASXL1* mutations frequently coexisted in CMML [17]. In addition, we found that the clonal evolution of *RUNX1* and/or *ASXL1* mutations occurred most frequently in CML with myeloid BC [18]. We had previously shown that the biological activities of RUNX1 mutants predicted sAML transformation from CMML and MDS [19]. Zhao et al. also found that RUNX1 mutants exhibited decreased transactivation activity as well as had a dominant-negative function on the WT-RUNX1 as a result of AML transformation in a subset of CML patients [20].

The present study was sought to demonstrate the biological and functional evidence for a collaborative association of RUNX1 mutant and ASXL1 mutant for myeloid transformation. We identified HIF-1α targeting a new pathway which may be critical for the leukemic progression of *RUNX1*/*ASXL1*-mutated myeloid malignancies.

## Materials and methods

### Patient samples and cell lines

Between 1991 and 2013, 104 adult patients were consecutively diagnosed as CMML according to the WHO classification at the Chang Gung Memorial Hospital (CGMH). Mutational analyses of *ASXL1* and *RUNX1* were performed as described previously [16, 21]. HL-60 cells were obtained from ATCC and the human leukemia cell lines, K562, THP-1, and U937, used from our stock and were authenticated by cellular morphology and STR analysis at CGMH (January–February 2017) and cultured in RPMI-1640 medium supplemented with 10% FBS, 2 mM L-glutamine, and 1× antibiotic-antimitotic in a humidified chamber with 5% CO_2_ atmosphere at 37 °C. Murine myeloid leukemia 32Dcl3 (32D) cells were cultured in the presence of 1 ng/mL murine-IL-3 under similar conditions. EcoPack2-293 cell lines were cultured in DMEM medium under identical conditions.

### Vector construction

The full-length cDNA of human *RUNX1-*WT with FLAG-tag was constructed into the NheI/NotI multiple cloning sites of lentiviral vector pCDH1-MSCV-MCS-EF1-Puro (pCMSCV, EV2) according to the standard method and verified by sequencing. Point mutant, R135T of *RUNX1* gene, was generated from FLAG-*RUNX1*-WT using site-directed mutagenesis (KAPA HiFi HotStart, Kapa Biosystems) and confirmed by full-length DNA sequencing. *ASXL1*-R693X tagged with a FLAG epitope at the N-terminus was subcloned into pIRES2-EGFP-vector, then either empty vector or FLAG-*ASXL1*-R693X-IRES2-EGFP cassette was inserted into the pCMSCV vector to make pCMSCV-IRES2-EGFP (EV1) or pCMSCV-FLAG-*ASXL1*-R693X-IRES2-EGFP plasmid. Similarly, *RUNX1*-R135T and *ASXL1*-R693X were constructed into the BglII/HpaI multiple cloning sites of retroviral vector pMSCVhyg and pMSCVneo plasmids respectively. All sequences were confirmed by direct sequencing before expression in cells. The pLKO.1-puro plasmid-based shRNAs, including sh*Luc* (luciferase shRNA, TRCN231719), human *ID1*-sh1 (TRCN0000019029), and *ID1*-sh2 (TRCN0000019030), were obtained from the National RNAi Core laboratory, Taiwan.

### Lentiviral and retroviral transduction

Lentivirus production and infection were performed as our previous description [19]. Plasmid DNAs of retroviral vector were transfected into EcoPack2-293 cells using Lipofectamine 2000. The supernatant of the transduced cells containing packaged retroviruses was collected at 48 h after transfection, centrifuged and concentrated with Retro-X Concentrator (Clontech) according to the manufacturer’s protocol. Cultured cells were spun infected in the presence of 8 μg/mL polybrene (Sigma-Aldrich) and cultured for 60 h. The infected cells were green fluorescent protein (GFP) sorted or subjected to drug selection, if necessary for 2 weeks to obtain stable clones.

### Induction of differentiation and cell proliferation assays

Phorbol 12-myristate 13-acetate (PMA)-mediated myelomonocytic differentiation of U937 cells and megakaryocytic differentiation of K562 cells were induced by applying 40 nM PMA (Sigma-Aldrich) dissolved in dimethyl sulfoxide (DMSO). For control, all samples were treated DMSO only and cultured under identical conditions. To induce granulocytic differentiation of 32D cells, they were treated with 50 ng/mL granulocyte colony-stimulating factor (G-CSF) (PeproTech) for 96 h. Before assaying for differentiation and proliferation, transduced 32D, K562, or U937 cells were subjected to drug selection with the selective drug(s), if necessary. After stable transduction of 32D cells with various plasmids, cells were washed using phosphate-buffered saline (PBS) twice and cultured in complete medium with 1 ng/mL IL-3 or without IL-3 for indicated times. Cell viability was assessed by manual counting using a hemocytometer following with trypan blue staining at different time points. Similarly, the cell growth of transduced K562 cells was measured. To check cell growth of transduced K562 and U937 cells in the presence of chrysin (Santa Cruz) (HIF-1α inhibitor), cells were incubated in the presence of 30 μM chrysin for 72 h.

### Flow cytometry analysis

Various differentiating inducing reagent-treated cells were collected after specific time incubation, washed in PBS, and counted. 5 × 10^5^ cells were second time washed in PBS containing 1% bovine serum albumin then incubated with CD11b PE (eBioscience) or anti-CD61-PerCP-Ab (BD Pharmingen) or for 30 min at room temperature. Fluorescence was measured by flow cytometry (BD AriaIII) using the BD Cell-Quest Pro version 4.0.1 software.

### Western blot analysis

Cell lysates were subjected to immunoblotting with the following antibodies: anti-RUNX1 (25315-1-AP) and anti-HIF-1α (20960-1-AP) obtained from ProteinTech; anti-histone-H3 (ab70550), anti-H3K4me3 (ab8580), anti-H3K27me3 (ab6002), and anti-AKT1 (phosphor S473, ab81283) from Abcam; anti-ID1 (sc-133104) and anti-ASXL1 (sc-85283) from Santa Cruz Biotechnology; anti-EZH2 (#4905) from Cell Signaling Technology; anti-AKT1+2+3 (GTX121937) from Gene Tex; and anti-flag (F3165) and anti-actin (A5441) from Sigma-Aldrich. Immunoprecipitation reaction was performed using transiently and stably expressed FLAG-RUNX1-WT/RUNX1-R135T into HEK293T and K562 cells respectively. For the endogenous interaction of ASXL1 and EZH2, we used K562 and U937 whole-cell extract. Cell lysates were subjected to immunoprecipitation with either anti-FLAG M2 affinity gel (A2220, Sigma) or Protein G Mag Sepharose Xtra (Blossom Biotenchnologies, Inc.) according to the manufacturer’s instructions. Either non-immune IgG or empty vector (EV) control was used as negative control. Cell lysate preparation and immunoblotting were performed as reported previously [22].

### Real-time quantitative PCR

Total RNA was extracted from frozen or cultured cells, patient’s bone marrow samples, and mouse spleen, liver, and BM using Trizol total RNA isolation reagent (Invitrogen). The sample was reversely transcribed to cDNA with random hexamers using the Superscript III First-Strand Synthesis System for RT-qPCR Kit (Invitrogen). The cDNA was used for quantitative PCR with iQTM SYBR® Green Supermix (BIO-Rad) according to the manufacturer’s protocol. The sequences of oligonucleotides used for quantitative PCR (qPCR) are listed in Additional file 1: Table S1. Samples were run in duplicate and transcript levels were calculated as 2(^−∆∆Ct^), and the threshold cycle number for different genes was normalized to the expression of GAPDH.

### Chromatin immunoprecipitation analysis

The ChIP assays were carried out according to the manufacturer’s (R&D Systems Inc. USA) protocol using Human/Mouse HIF-1α Chromatin Immunoprecipitation Kit (Cat. No. ECP1935). Cross-linked chromatin was incubated overnight at 4 °C with the anti-HIF-1α antibody on a rotating device. Immunoprecipitated DNA and input samples were cleaned up using a DNA purification kit. Purified ChIP product was quantitated by real-time qPCR using SYBR Green on an ABI Prism 7900HT Fast Real-Time PCR system. RT-qPCR quantification of ChIP was performed in duplicate using primers specific for promoter regions. ChIP was quantified relative to inputs. The following primers were used for quantitative ChIP-PCR:

*ID1* (F): ACACGAACAGCAACATTATTTAGGAA, *ID1* (R): GAGGCCCGAACGGAGAAG.

*VEGF* (F): TTGATATTCATTGATCCGGGTTT, *VEGF* (R): TCTTGCTACCTCTTTCCTCTTTCTG.

*GAPDH* (F): CTTGACTCCCTAGTGTCCTGCT, *GAPDH* (R): CCTACTTTCTCCCCGCTTTTT.

### Gene expression microarray analysis

Gene expression analysis was carried out using Affymetrix Human Gene U133 Plus 2. Total RNA was extracted from stably transduced K562 cells using the Trizol reagent method. Amplification and biotin labeling of fragmented cDNA was carried out using the standard Affymetrix protocol. Labeled probes were hybridized to the Affymetrix GeneChip Hybridization Oven 645 and GeneChip Fluidics Station 450 and scanned. Expression data were extracted from image files produced on GeneChip Scanner 3000 7G. The scanned images were analyzed with the Standard Affymetrix protocol. GeneChip analysis data normalized with RMA by Affymetrix Expression Consol Ver 1.4 (EC 1.4) software and fold change were calculated compared to the empty vector control. The upregulated genes were selected using the criteria of undergoing a > 2.0-fold change in gene expression. The gene expression microarray data have been deposited in the Gene Expression Omnibus (GEO) database with accession number GSE99640.

### Mice and BMT experiment

All animal experiments were approved by the Department of Animal Experimentation at CGMH. C57BL/6 mice (NARlabs, Taiwan) were used for BMT experiments. Mouse BMT was performed as described previously [23]. Briefly, BM cells were isolated from 8- to 12-week-old mice which were pretreated with 5-fluorouracil (150 mg/kg) 4 days before the operation. BM cells were cultured with RPMI containing 20% FBS, 2 mM L-glutamine, 1× antibiotic-antimitotic, 100 ng/mL mouse stem cell factor, and 10 ng/mL mouse IL-3. The primary murine BM cells then were transduced with retroviruses by spin inoculation in the presence of 8 μg/mL polybrene. The infection was repeated after 48 h of the first infection, and transduced BM cells (1 × 10^6^ cells/mouse) were transplanted into intraperitoneally injected busulfan (a single dose of 30 mg/kg before 3 days) mice via the tail vein [24, 25]. Mice were monitored every day, and moribund mice were euthanized according to the animal house guideline.

### Mouse bone marrow colony-forming assay

Colony-forming assays were performed according to the manufacturer’s instructions (MethoCult M3434; StemCell Technologies, Vancouver, BC, Canada). Briefly, 2 × 10^4^ transduced mouse BM cells were mixed with 2 mL MethoCult medium in duplicate cultures in 6-well tissue-cultured plate. Colonies of more than 50 cells were scored on day 8 of cultures. Serial replating assays were performed on day 8 of the previous culture. All cells were harvested, washed twice with RPMI medium, and counted. A similar number of cells were then replated, and the process was repeated for four times to check colony formation and self-renewal activity. Results from colony-forming unit (CFU) assays to assess granulocyte (CFU-G; colorless, more dense, smaller and round cells), macrophage (CFU-M; colorless, less dense, large and elongated cells), granulocyte with macrophage (CFU-GM; colorless, heterogeneous population of small, round cells and large, elongated cells) and erythroid (CFU-E; red color either very small colonies or BFU-E; clusters containing group of tiny cells in irregular shape).

### Luciferase reporter assay

K562 cells were co-transfected with luciferase reporter plasmid of HRE promoter, pGL4.42[Luc-2p/HRE] (Promega, RE4001) combined with EV, RUNX1-WT, and RUNX1-R135T plasmids, and 32D cells with EV, ASXL1-R693X, RUNX1-R135T, and ASXL1-R693X + RUNX1-R135T; pEGFP-C1 (Promega) was used as internal control using TransIT-2020 transfection reagent (Mirus, Blossom Biotechnologies Inc.). Forty-eight hours later, cells were incubated with 100 μM CoCl_2_ (Alfa Aesar, B22031) for an additional 24 h. After 72 h, cells were harvested, lysed with passive lysis buffer (Promega), and mixed with Bright-Glo Luciferase assay reagent (Promega) for detection of luciferase activities and GFP fluorescent intensity. The relative activity of each sample was derived by normalization of each luciferase intensity with its GFP intensity and then divided by the value of EV.

### Statistical analysis

The Kaplan–Meier analysis was used to evaluate survival. Differences in survival were assessed using the log-rank test. All in vitro data represented here were mean ± SD as indicated. In all analyses, *P* values were two-tailed, and values < 0.05 were considered significant for all analyses.

## Results

### Expression of *RUNX1* mutant augmented cell proliferation and impaired differentiation of *ASXL1*-mutated cells

**Fig. 1:**
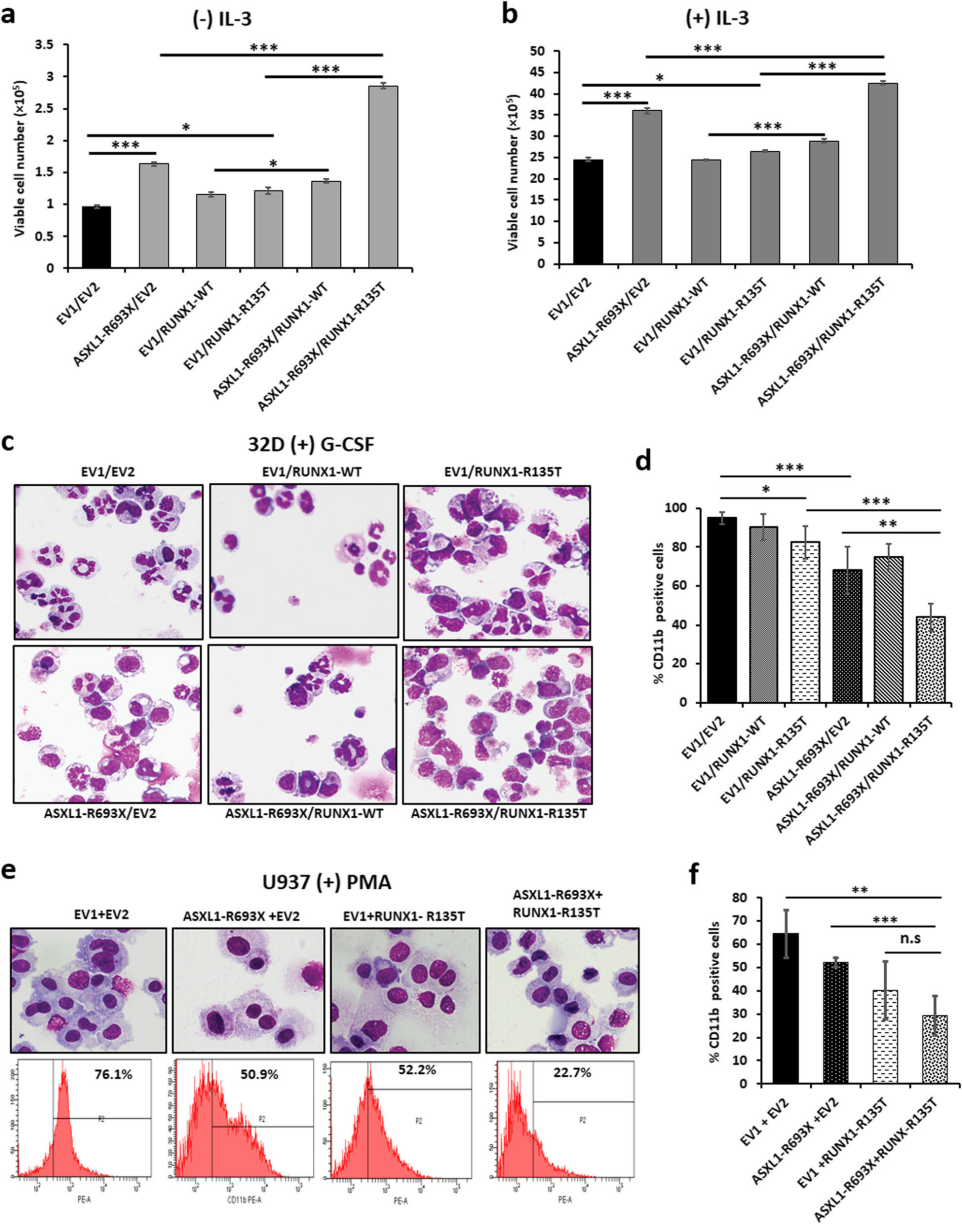
The collaboration of ASXL1 and RUNX1 mutants augmented proliferation and impair differentiation in vitro. **a**, **b** IL-3-dependent murine myeloid 32D cells stably transduced with ASXL1-R693X, RUNX1-WT/R135T, and combination of ASXL1-R693X and RUNX1-R135T were cultured in the absence (**a**) or in the presence (**b**) of IL-3 for 96 h. The number of viable cells is shown. **c**, **d** The stably transduced 32D cells were cultured with 50 ng/mL of G-CSF for 5 days; cell morphology with Wright–Giemsa-stained smear, original magnification × 400 (**c**), and percentage of CD11b-positive cells are shown (**d**). **e**, **f** The stably transduced U937 cells were cultured with 40 nM of PMA for 96 h; cell morphology with Wright–Giemsa-stained smears (original magnification × 400) (**e**, upper panel), representative flow cytometry analyses (**e**, lower panel), and percentage of CD11b-positive cells (**f**) are shown. All data error bars in **a** and **b** represented here are the mean ± SD of duplicate cultures and repeated three times; error bars in **d** and **f** represented the mean ± SD of four and three independent experiments respectively. *P < 0.05, **P < 0.03, ***P < 0.01; n.s., not significant

To understand the biological significance of RUNX1-MTs with ASXL1-MTs in CMML patients, we first examined the effect of RUNX1 mutant on cell proliferation and differentiation in *ASXL1*-mutated cells. As deletion or frameshift deletion of *ASXL1* and runt domain mutations of *RUNX1* was very common in CMML and CML with myeloid BC patients, we selected *RUNX1*-R135T and *ASXL1*-R693X mutations for the functional study. We overexpressed *ASXL1*-R693X, *RUNX1*-WT/R135T, or combination of *ASXL1*-R693X with *RUNX1*-R135T in IL-3-dependent murine 32D myeloid cells, and expressions were checked by using both qRT-PCR and immunoblot analyses (Additional file 1: Figure S1a-c). The results showed that expression of RUNX1-R135T in ASXL1-R693X-mutated clone enhanced cell proliferation and growth of 32D cells induced by IL-3 free or in the presence of IL-3 (Fig. 1a, b, Additional file 1: Figure S1d). We observed that the collaboration of ASXL1-R693X with RUNX1-R135T reduced G-CSF-induced myeloid differentiation compared to either ASXL1-R693X or RUNX1-R135T-expressing 32D cells (Fig. 1c, d) as evidenced by an increased in more mature neutrophils (Fig. 1c) and the number of CD11b-expressing 32D cells (Fig. 1d). Similarly, PMA-induced monocytic differentiation of U937 cells, a human leukemia monocyte cell line, was also attenuated by the combined expression of RUNX1-R135T with ASXL1-R693X compared to single mutant (Fig. 1e, f, Additional file 1: Figure S2a). In contrast, transformed U937 control cells with the treatment of DMSO only did not affect (Additional file 1: Figure S2b).

### Biologically *RUNX1*-R135T affected *ASXL1*-Y591Y/X mutant K562 cells

**Fig. 2:**
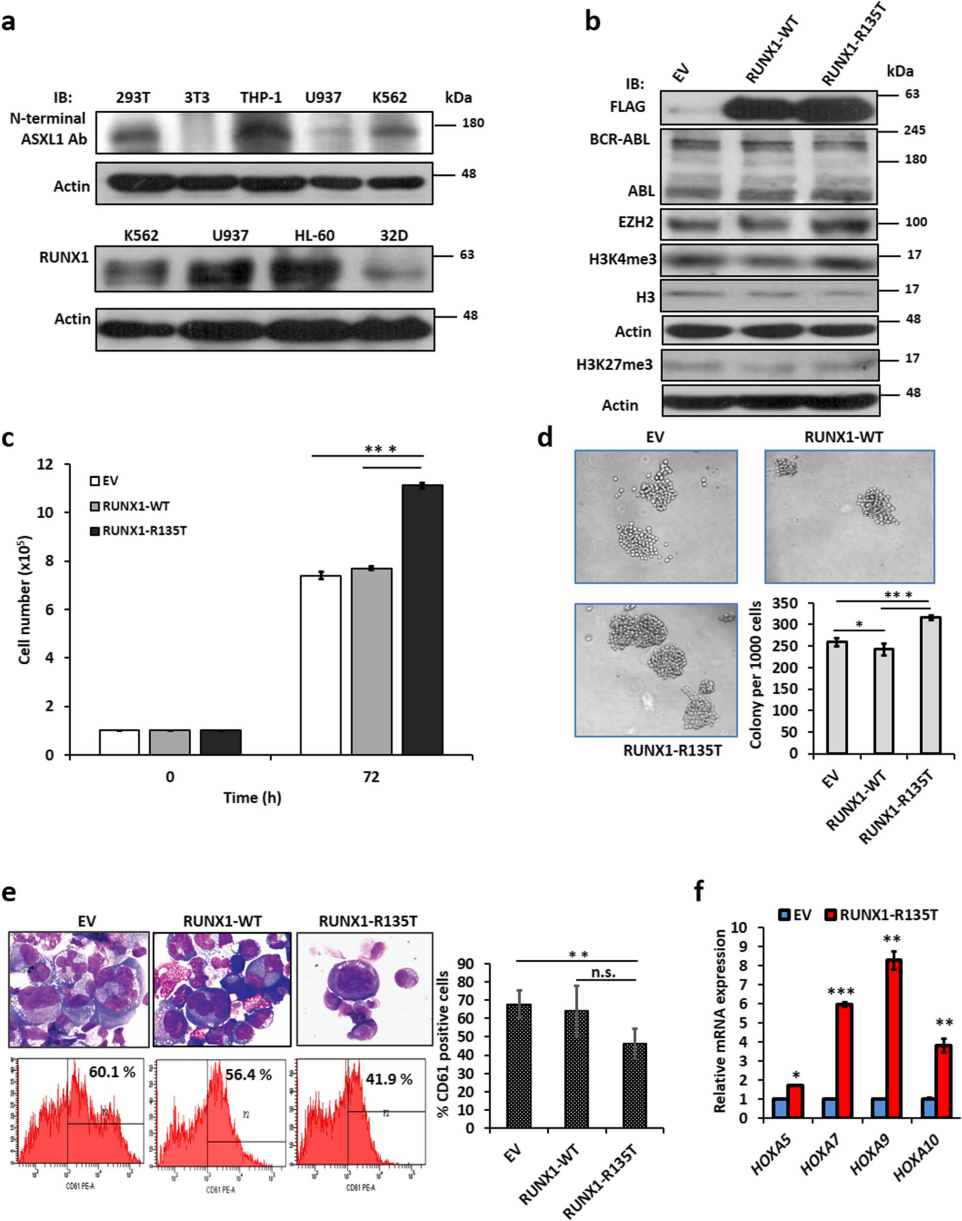
RUNX1-R135T cooperatively affected *ASXL1* mutant K562 cells. **a** Endogenous expression of ASXL1 and RUNX1 in different leukemia cell lines including the *ASXL1*-mutated K562 cell line. **b** Stably transduced RUNX1-WT/MT in K562 cells and showing BCR-ABL, ABL, EZH2, and histone-modifying protein expression in transformed cells by immunoblot analysis. **c**, **d** K562 cells were stably transduced with WT- and RUNX1-R135T mutants, then cell proliferation was checked by trypan blue exclusion method (**c**) and colony formation ability was assayed in methylcellulose containing complete RPMI medium (**d**). After 10 days, colonies were photographed and counted (original magnification × 100). **e** Cell morphology analyzed by modified Wright–Giemsa-stained smears after treatment with 40 nM PMA in EV, RUNX1-WT, and RUNX1-R135T-transduced K562 cells for 96 h, magnification × 400 (left, upper panel), and digital images were acquired using Olympus (model no. U-TV0.5XC-3) microscopes equipped with a digital camera. Flow cytometry analyses of megakaryocytic marker (CD61) after the treatment of PMA in transduced K562 cells; a representative experiment is shown (left, lower panel); the percentage of CD61 expressing K562-positive cells are shown (right). **f** Analysis of mRNA levels of *HOXA5*, *HOXA7*, *HOXA9*, and *HOXA10* by RT-qPCR after overexpression of RUNX1-R135T and EV in K562 cells. Error bars represent mean ± SD based on three independent experiments. *P < .05, **P < .01, ***P < .001, either compared with the control or as indicated in figures; n.s., not significant

We and others found that the human CML cell line, K562, is carrying Philadelphia chromosome harboring *ASXL1* mutation (Y591Y/X). We checked the endogenous ASXL1 and RUNX1 protein expression in different cell lines and found that both proteins are expressed in K562 cells (Fig. 2a). However, we could not detect mutant ASXL1 protein in K562 cell due to the high background and several non-specific signals. Though K562 cell line harbored heterozygous mutation of *ASXL1*, interestingly, K562 cells expressed endogenous H3K27me3, and the immunoprecipitation studies with endogenous ASXL1 protein in K562 cells was co-immunoprecipitated with endogenous EZH2 (Additional file 1: Figure S3a and b). To check the effect of *RUNX1*-R135T in *ASXL1*-mutated cells on the leukemogenesis, we stably overexpressed *RUNX1*-WT and *RUNX1*-R135T in K562 cells. The expressions of RUNX1-WT/MT in K562 cells were confirmed using immunoblot analysis (Fig. 2b). As K562 cells are BCR-ABL1 positive, we checked the fusion protein BCR-ABL1 and ABL1 expression in transformed K562 cells. The results showed that either BCR-ABL or ABL expression did not change in RUNX1-R135T-expressing K562 cells compared to EV or RUNX1-WT cells. We also found that EZH2 and H3K4me3 increased modestly in RUNX1-R135T cells (Fig. 2b); however, H3K27me3 was not changed in mutant cells. We observed that cell proliferation and colony formation ability were augmented in K562 cells overexpressed with RUNX1-R135T (Fig. 2c, d). Moreover, RUNX1-R135T impaired PMA-induced megakaryocytic differentiation of K562 cells (Fig. 2e). The expression of CD41 and CD61 at the cell surface is used as a hallmark of megakaryocytic differentiation [26, 27]. We found that CD61 expression was much higher than CD41 expression upon stimulation of PMA in K562 cells [28]. Empty vector control and RUNX1-WT expressing K562 cells exhibited enlarged and multilobed nucleus, which were detected by Wright-stained smear (Fig. 2e, upper) whereas RUNX1-R135T-expressing K562 cells had shown immature cell morphology. Flow cytometry analyses with CD61 antibody also confirmed the cooperative role of *ASXL1* mutation and *RUNX1*-R135T on megakaryocytic differentiation, with CD61 expression being reduced from 60.1% in PMA-treated control K562 cells to 41.9% in *RUNX1*-R135T-expressing K562 cells (Fig. 2e, lower and right panel). In contrast, transformed K562 control cells treated with DMSO alone did not affect CD61 expression (Additional file 1: Figure S3*c*). Here, we also observed that RUNX1-R135T upregulated *HOXA5*, *HOXA7*, *HOXA9*, and *HOXA10* in *ASXL1* mutant K562 cells (Fig. 2f).

### The collaboration of RUNX1-R135T with ASXL1-R693X enhanced clonogenic and self-renewal ability of mouse bone marrow cells

**Fig. 3:**
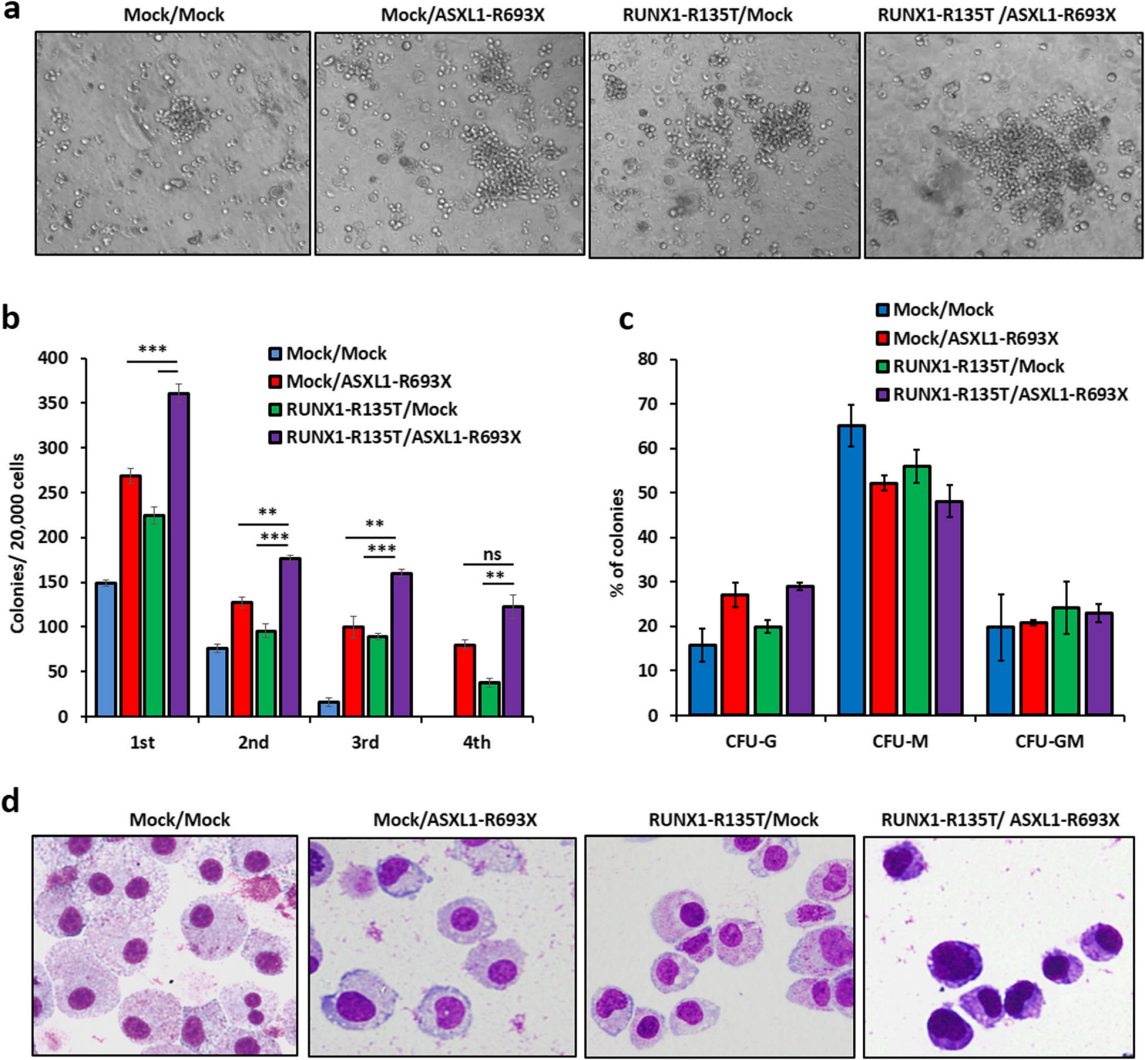
Cooperation of ASXL1-R693X and RUNX1-R135T in mouse BM cells increased myeloid colony. **a**, **b** Colony-forming potentials of mouse BM cells transduced with indicated plasmids in methylcellulose colony-forming media; representative images of colonies in the second round plating were shown. Colonies were photographed and counted manually, original magnification × 100. Columns represent the number of serially replated colonies. **c** The proportion of CFU colonies of second-round serial replating. **d** Cytospin smeared preparations of cultured cells in the first plating colony assays by Wright–Giemsa staining was shown. Error bars represent the mean ± SD from duplicated cultures. *P < 0.05, **P < 0.03, ***P < 0.01

To determine the collaborative effect of ASXL1-R693X and RUNX1-R135T on colony formation and self-renewal activity in murine bone marrow cells (BMC), we retrovirally transduced *ASXL1*-R693X and *RUNX1*-R135T mutant and the combination of *ASXL1*-R693X/*RUNX1*-R135T, including empty vector constructs. We examined their colony-forming ability (Fig. 3a) and self-renewal activity (Fig. 3b) and analyzed CFU-G, CFU-M, and CFU-GM (Fig. 3c) in M3434 methylcellulose-based medium. However, we could not detect erythroid (CFU-E) and burst-forming unit erythroid (BFU-E) colonies. We found that *RUNX1*-R135T/*ASXL*1-R693X-transduced BM cells produced significantly more colonies with relatively immature morphology (Fig. 3d) compared with either mutant or empty vector control BM cells.

### The collaboration of RUNX1-R135T with ASXL1-R693X in the myeloid transformation

**Fig. 4:**
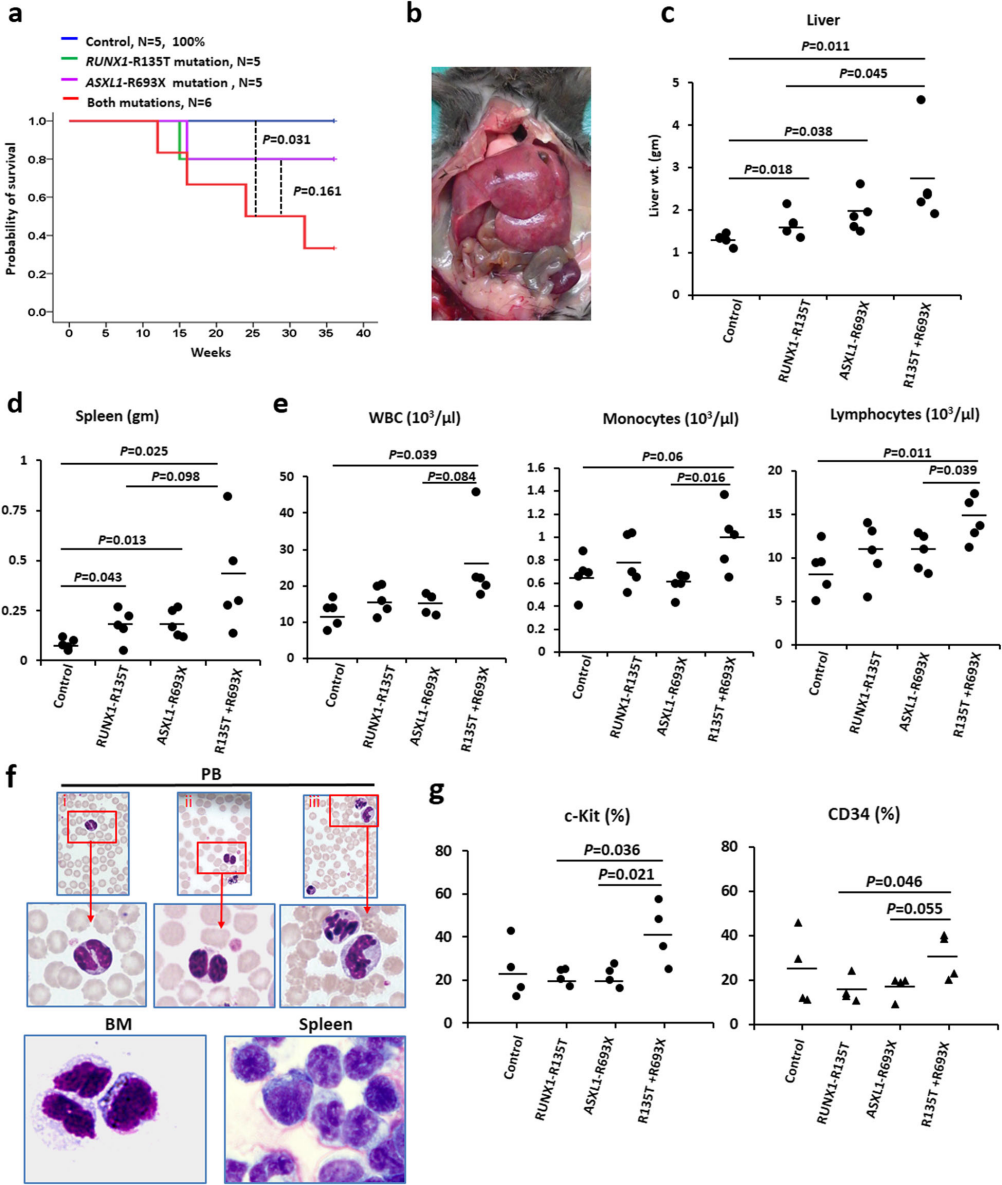
ASXL1-R693X collaborates with RUNX1-R135T in myeloid transformation. **a** Kaplan–Meier curve shows the survival of mice transplanted with BM cells transduced with empty vector, ASXL1-R693X, RUNX1-R135T, and combined expression of ASXL1-R693X and RUNX1-R135T. *P* values were calculated using a log-rank test. **b** Significant hepatomegaly and splenomegaly in diseased mouse transplanted with BM cells transduced with ASXL1-R693X and RUNX1-R135T. A representative photograph is shown. **c**, **d** Quantification of hepatomegaly (**c**) and splenomegaly (**d**) in mice transduced with EV, ASXL1-R693X, RUNX1-R135T, and coexisted mutations of ASXL1 and RUNX1 are shown (*n* = 5). **e** Peripheral blood counts of transduced mice (*n* = 5 for each group). **f** Representative peripheral blood smears from mice transduced with both ASXL1-R693X and RUNX1-R135T showing dysplastic neutrophils (i–ii) and hypersegmented nuclei (iii) (upper panel, magnification × 400); BM and spleen cytospin smears showing abnormal myeloid cells (lower panel, magnification × 400). **g** Flow cytometric analyses of myeloid progenitors in BM cells derived from mice transplanted with ASXL1-R693X, RUNX1-R135T, combined expression of ASXL1-R693X and RUNX1-R135T, and EV control (*n* = 4 of each group). *P* value showing calculated as indicated in the figures

To explore the in vivo transformation effect of simultaneous expression of *ASXL1*-R693X and *RUNX1*-R135T, we carried out bone marrow transplantation (BMT) experiments using murine BMC retrovirally transduced with either *ASXL1*-R693X or *RUNX1*-R135T mutant and the combination of *ASXL1*-R693X/*RUNX1*-R135T, including empty vector constructs. To determine whether susceptibility to the development of hematologic malignancy is affected by co-expression of *ASXL1* and *RUNX1* mutations, we monitored mice up to 9 months. Mice carrying an empty vector displayed no hematologic abnormalities and had a normal life span. At 9 months post-transplantation, four of six mice transplanted with BM cells expressing both *ASXL1*-R693X and *RUNX1*-R135T mutants died with marked splenomegaly and hepatomegaly, and a short median survival of 24 weeks compared with one of the five mice transplanted with BM cells expressing either *ASXL1* or *RUNX1* mutant died with a medium latency of 37.2 weeks (Fig. 4a–d). Peripheral blood counts showed leukocytosis in combined-mutated mice, but no thrombocytopenia was observed (Fig. 4e; Additional file 1: Figure S4a). Morphologic examination of peripheral blood smears, BM and spleen cytospin prepared smears from transplanted mice with combined expression of *ASXL1* and *RUNX1* mutants, showed morphological abnormalities at 9 months post-transplantation including hyposegmented (bilobed) neutrophils with fine nuclear bridging consistent with pseudo-Pelger-Hűet anomaly, hypersegmented neutrophils, and immature cells (Fig. 4f). Flow cytometry analyses of BMC revealed more immature cells and high expression of CD11b and Gr-1 in diseased mice compared to control (Fig. 4g, Additional file 1: Figure S4b-d).

### Acquisition of RUNX1-R135T increased ID1 expression in vitro and in vivo

**Fig. 5:**
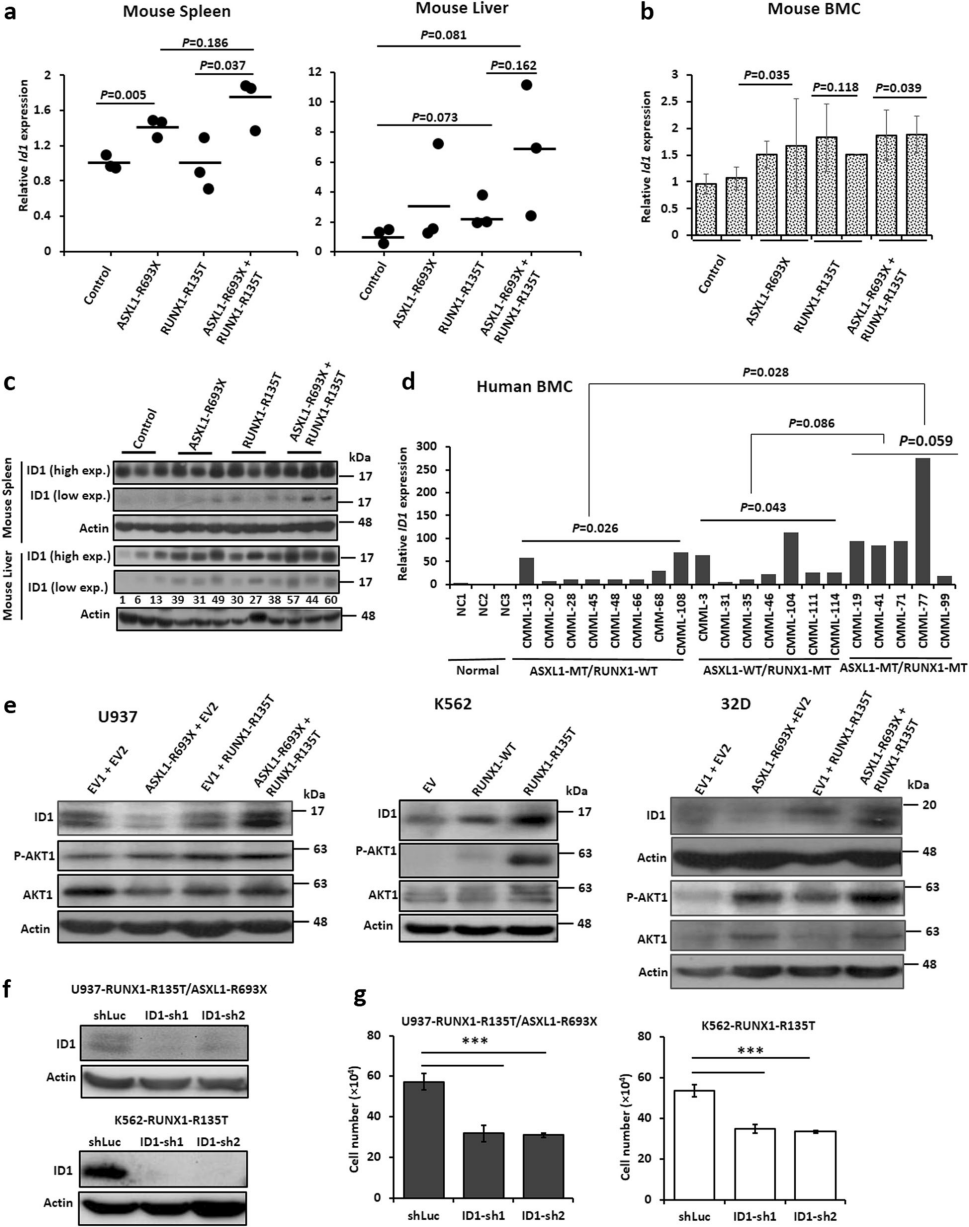
Collaborative mutations of *ASXL1* and *RUNX1* enhanced ID1 expression. **a**, **b** mRNA expression of *Id1* was analyzed by RT-qPCR in the spleen (**a**, left, *n* = 3), liver (**a**, right, *n* = 3), and BMC (**b**, *n* = 2) from killed or moribund mice transplanted with control, *ASXL1*-R693X, *RUNX1*-R135T, and combined mutations of *ASXL1* and *RUNX1*. Error bars represent the mean ± SD of two repetitions. **c** Immunoblot analyses of ID1 in the liver and spleen from BMT-mice. **d** Relative mRNA expression of *ID1* in BM leukemic cells from patients harboring either *ASXL1*-MT (*n* = 8) or *RUNX1*-MT (*n* = 7) and coexisted *RUNX1* and *ASXL1* mutations (*n* = 5). Gene expression was measured using real-time qPCR and shown as a relative to normal bone marrow cells (average of 3). NC indicated normal controls. **e** Immunoblot analyses with indicated antibodies in transduced U937 cells (left), K562 cells (middle), and 32D cells (right); β-actin representing as an internal control. **f** Knocked down *ID1* in the transduced U937 and K562 cells using two different shRNA and scrambled (shLuc), and silencing efficiency was checked by immunoblot analyses after 5 days. **g** Silenced cells were incubated for 72 h, and cell proliferation was measured by trypan blue exclusion method. Data represented here are the mean ± SD of triplicate cultures, and experiments repeated twice. *P* value showing in the figure calculated either compared with the control or as indicated in figures. The values (**c**) indicating relative signal density corresponding to actin expression

We performed a gene expression profile of K562 cells transduced with EV, *RUNX1*-WT, and *RUNX1*-R135T mutation. Gene expression profile revealed that 147 genes were upregulated more than twofold in *RUNX1*-R135T-expressing K562 cells compared to EV control cells (Additional file 2: Dataset S1). From gene expression data analysis, we found that the inhibitor of DNA binding 1 (*ID1*), a key transcriptional regulator of HSC (hematopoietic stem cell) lineage commitment [29], was upregulated in *RUNX1*-R135T-transduced K562 cells compared to EV and *RUNX1*-WT-expressing cells. To elucidate the molecular mechanism for disease transformation induced by the collaboration of ASXL1-MT and RUNX1-MT, we analyzed the expression of *ID1* at the mRNA level in different myeloid cell lines transduced with *ASXL1*-R693X, *RUNX1*-R135T and co-expression of *ASXL1*-R693X and *RUNX1*-R135T (Additional file 1: Figure S5a). To evaluate if *Id1* overexpression correlates with the development of leukemia in mice, we examined *Id1* expression in the spleen, liver, and BM of mice induced by *ASXL1*-R693X, *RUNX1*-R135T, and combination of *ASXL1* and *RUNX1* mutants as well as control. We found that the mRNA level of *Id1* was increased in mice transduced by both *ASXL1* and *RUNX1* mutants versus those transduced by *ASXL1*-R693X or *RUNX1*-R135T alone (Fig. 5a, b). Similarly, the ID1 protein level was increased in the liver and spleen of mice transduced with either mutation or combined mutations of *ASXL1* and *RUNX1* (Fig. 5c) compared to control. Moreover, we found that *ID1* expression was increased in clinical samples of BMC from patients with CMML at diagnosis harboring both *ASXL1* and *RUNX1* mutations compared to either *ASXL1* or *RUNX1* mutations (Fig. 5d). Increase of ID1 in different cell lines transduced with coexisted mutations of *ASXL1* and *RUNX1* correlated with the upregulation and activation of AKT1 signaling, although the expression of three proteins was dependent on different cell context manners (Fig. 5e). To check the requirement of ID1 in the context of *RUNX1*-MT/*ASXL1*-MT leukemogenesis, we knocked down *ID1* from transduced-U937 and K562 cells by using two different shRNAs. The results showed that the inhibition of ID1 decreased cell proliferation significantly (Fig. 5f, g). Similarly, inhibition of ID1 using pimozide, a reported ID1 inhibitor [30] on the transduced K562 cells, significantly reduced cell proliferation (Additional file 1: Figure S5b).

### RUNX1-R135T augmented HIF-1α and its target gene

**Fig. 6:**
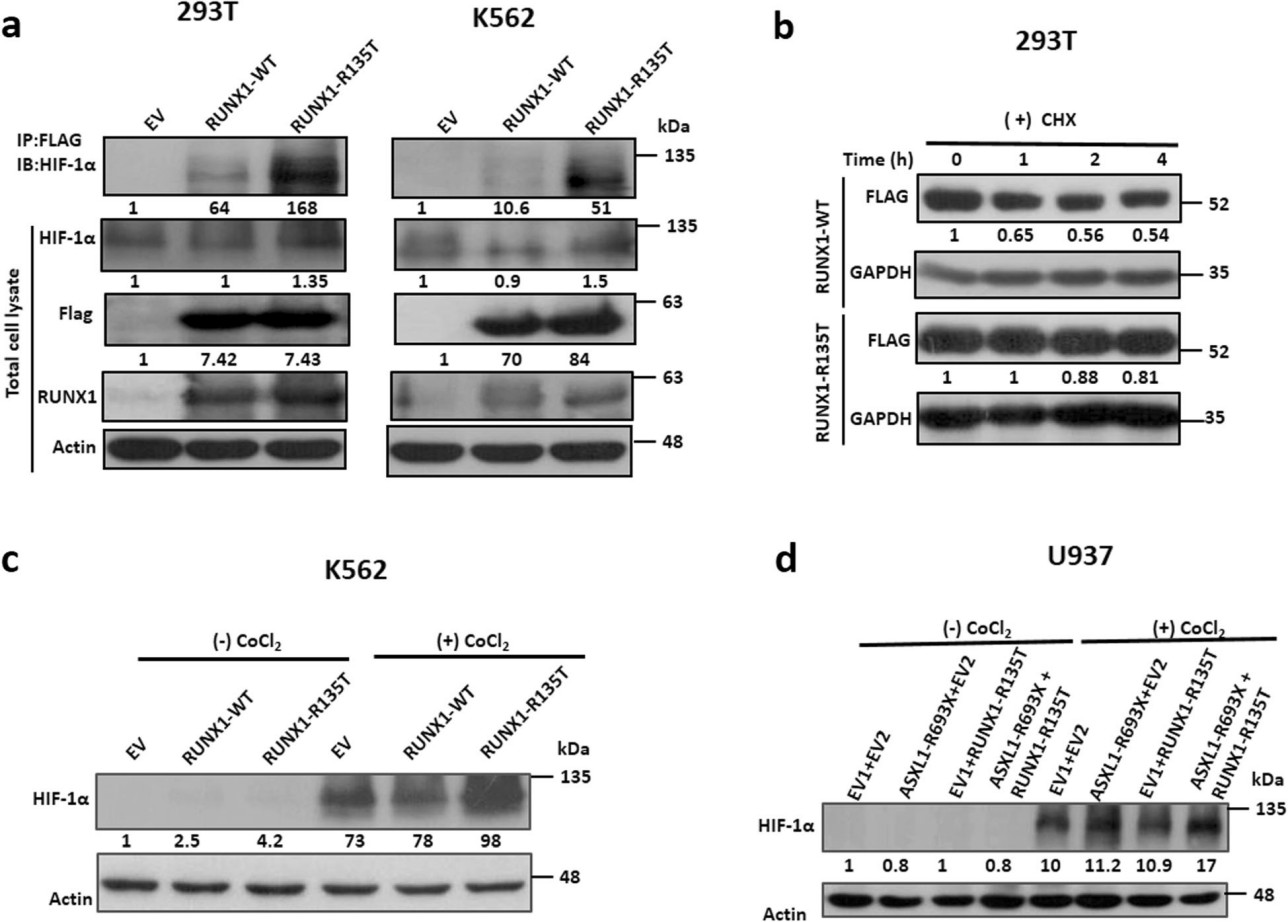
RUNX1-R135T mutation interacted more with HIF-1α and enhanced HIF-1α expression in ASXL1-R693X-mutated leukemia cells. **a** Transiently expressed FLAG-tagged RUNX1-WT, RUNX1-R135T, and EV control in HEK293T cells or stable-expressed in K562 cells treated with 100 μM CoCl_2_ for 24 h; immunoprecipitation was performed using anti-FLAG M2 affinity gel, and immunoblot was performed using HIF-1α, RUNX1, and FLAG antibody. β-actin representing as a loading control. **b** After transient expression of FLAG-tagged WT and mutant RUNX1 in HEK293T cells, cycloheximide (CHX) (100 μg/mL) was treated for different times. Cell lysates were immunoblotted with antibodies against indicated proteins. **c**, **d** After stable expression of RUNX1-WT and RUNX1-R135T in K562 cells (**c**) or ASXL1-R693X, RUNX1-R135T and coexisted expression of ASXL1-R693X and RUNX1-R135T including control in U937 cells (**d**) were treated with 100 μM CoCl_2_ or no CoCl_2_ for 24 h and checked HIF-1α expression by immunoblot analyses. The values in the immunoblot data indicating relative signal density corresponding to Actin or GAPDH expression. All signals from immunoprecipitation were standardized relative to the signal from EV control

**Fig. 7:**
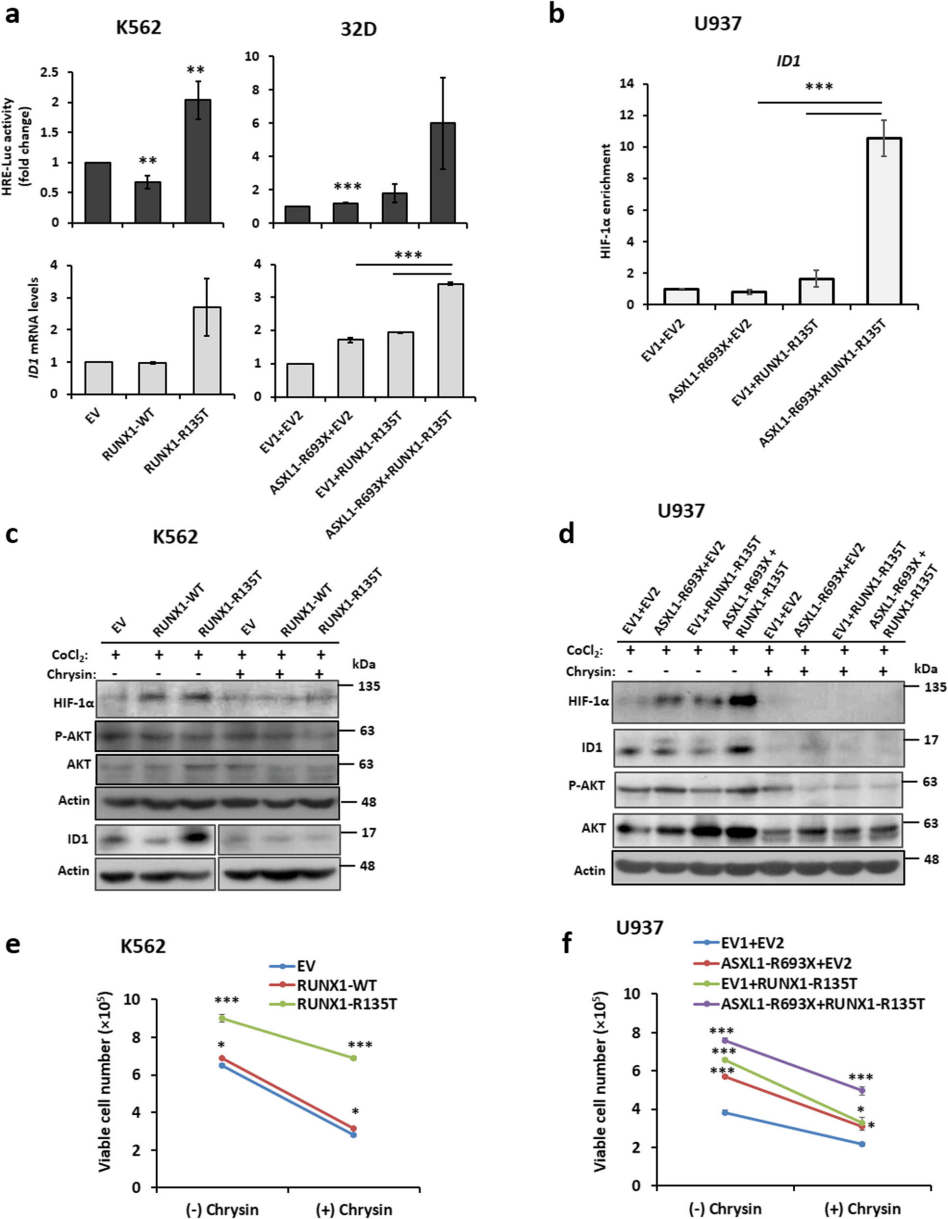
The collaboration of RUNX1-R135T with ASXL1-R693X in the enhancement of HIF-1α and its target gene. **a** K562 cells were co-transfected with luciferase reporter plasmid of HRE promoter, pGL4.42[Luc-2p/HRE], pEGFP with EV, RUNX1-WT, and RUNX1-R135T plasmids; 32D cells with EV, ASXL1-R693X, RUNX1-R135T, and ASXL1-R693X + RUNX1-R135T. Forty-eight hours later, cells were incubated with 100 μM CoCl_2_ for an additional 24 h. Increased folds of the relative HRE-Luc activities, which were normalized by GFP fluorescent intensity, were calculated against EV control (upper), and relative inhibitor of DNA binding 1 (*ID1*) mRNA levels was determined by real-time quantitative RT–PCR (lower). **b** Chromatin immunoprecipitation (ChIP) analyses were performed using the HIF1-α antibody in U937 cells overexpressed with ASXL1-R693X, RUNX1-R135T, ASXL1-R693X + RUNX1-R135T, and EV. *ID1* expression was measured using real-time qPCR, and ChIP-qPCR data is displayed as enrichment relative to the input. All signals from transformed-U937 cells were standardized relative to the signal from control U937 cells, which were set to 1.0. Error bars represented mean ± SD based on two independent experiments. **c**, **d** Transformed K562 (**c**) and U937 cells (**d**) were cultured with 30 μM chrysin or no chrysin for 2 h. CoCl_2_ (100 μM) was added, and the cells were incubated for another 12 h. The cells were harvested, lysed, and immunoblotted with indicated antibodies. **e**, **f** K562 cells were stably transduced with RUNX1-WT, RUNX1-R135T mutant, control (**e**), and U937 cells with ASXL1-R693X, RUNX1-R135T, and ASXL1-R693X + RUNX1-R135T including control (**f**). Cells were incubated in the presence of 30 μM HIF-1α inhibitor, chrysin, for 72 h, and viable cells were counted. Data represented here are the mean ± SD of duplicate cultures and experiments repeated three times. *P < .05, **P < .01, ***P < .001, compared with the control or as indicated in figures

**Fig. 8:**
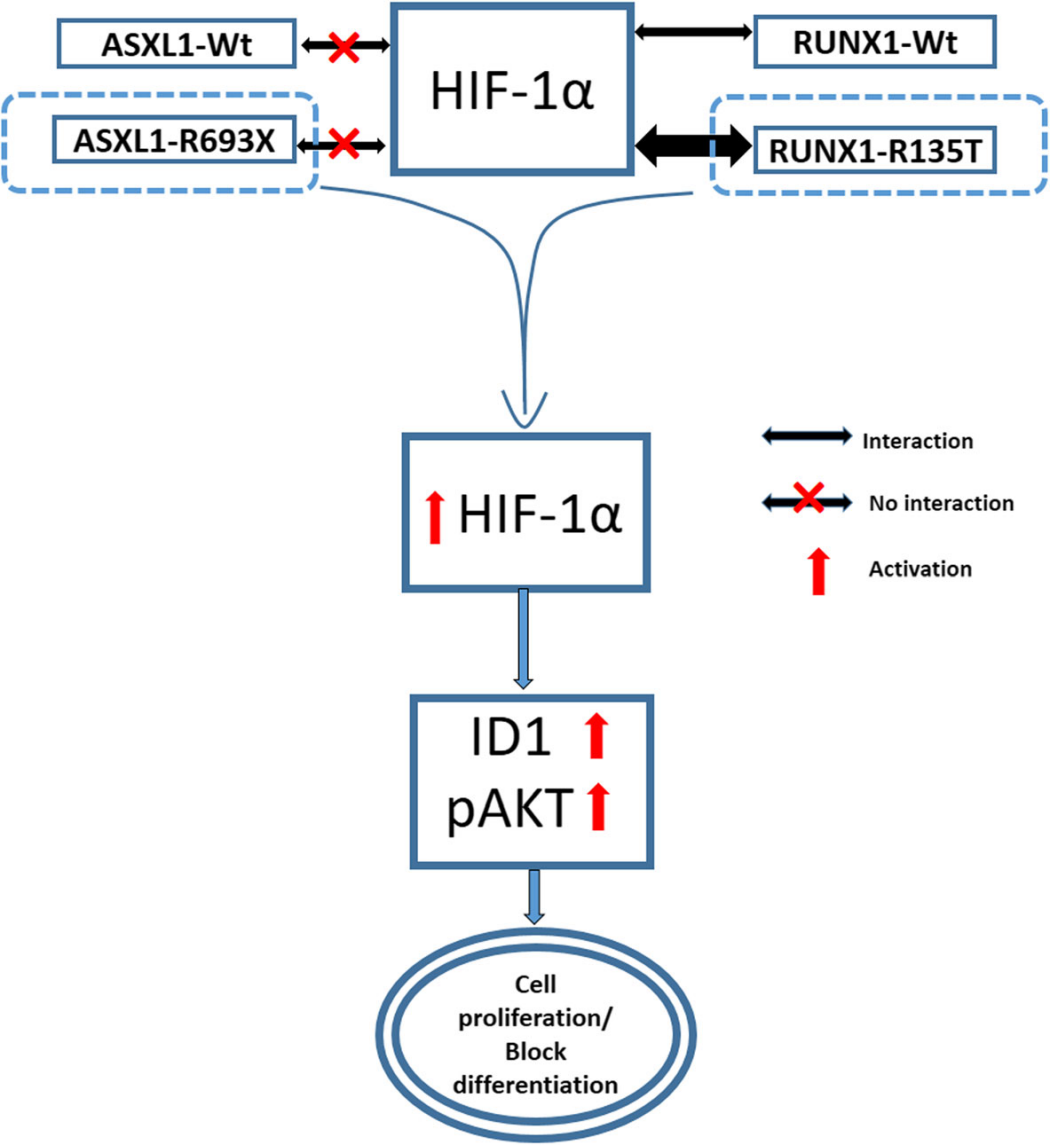
Schematic presentation of the crucial role of RUNX1-MT in the leukemogenesis of myeloid malignancies in *ASXL1*-mutated leukemia. Co-expression of ASXL1-R693X and RUNX1-R135T increased HIF1-α activity and its target gene, ID1 expression, enhancing cell proliferation, blocking differentiation, and promoting leukemogenesis

The previous study demonstrated that RUNX1 and HIF-1α directly interacted with each other, in which the runt homology domain of RUNX1 was mainly involved [31]. To determine whether RUNX1-R135T interacts with HIF-1α, we performed co-IP analysis using FLAG-tagged RUNX1-WT and RUNX1-R135T mutant-expressed in HEK293T and K562 cells in the presence of hypoxia-mimetic reagent CoCl_2_ at 100 μM. We found that RUNX1-R135T protein interacted with HIF-1α protein with a greater extent than RUNX1-WT protein (Fig. 6a). In addition, we could not find the interaction of ASXL1 protein either with RUNX1 or HIF1-α (Additional file 1: Figure S6a-c). We also found that RUNX1-R135T protein was more stable than RUNX1-WT protein (Fig. 6b and Additional file 1: Figure S7). Moreover, overexpression of RUNX1-R135T in K562 cells or combined expression of ASXL1-R693X and RUNX1-R135T in U937 cells enhanced HIF-1α expression (Fig. 6c, d). Then, we explored whether RUNX1-R135T affected the transcriptional function of HIF-1α protein by detecting hypoxia-responsive element (HRE)-driven luciferase (Luc) activity and mRNA levels of HIF-1α-targeted gene *ID1*. The results showed that the stabilization of HIF-1α by CoCl_2_ significantly increased the relative HRE-Luc activity by RUNX1-R135T overexpression and simultaneously elevated the mRNA level of *ID1* (Fig. 7a). We then asked whether either ASXL1-R693X and RUNX1-R135T or coexisted mutations of ASXL1-R693X and RUNX1-R135T affected the status of HIF-1α at the locus of *ID1*. ChIP assays were performed with antibodies against HIF-1α. Immunoprecipitated DNA was then subjected to PCR assays using primers in the promoter regions of *ID1*. Vascular endothelial growth factor (VEGF) was considered as a positive control of HIF-1α targeted genes. ChIP-qPCR for HIF-1α in U937 cells carrying *ASXL1*-R693X, *RUNX1*-R135T, and both mutations including the control revealed an enhancement of HIF-1α enrichment at the *ID1* and *VEGF* promoter regions with coexisted mutations of *ASXL1* and *RUNX1* compared to control or either mutation (Fig. 7b and Additional file 1: Figure S8). To elucidate the HIF-1α-targeted ID1 signaling, transduced K562 and U937 cells were incubated with 30 μM chrysin, a potent HIF-1α inhibitor [32], for 2 h and then treated with 100 μM CoCl_2_ for 12 h. Immunoblot data showed that inhibition of HIF-1α by chrysin significantly reduced ID1 expression with a modest reduction of AKT1 signaling (Fig. 7c, d). Furthermore, treatment of transduced K562 and U937 cells with 30 μM chrysin for 72 h reduced cell proliferation significantly (Fig. 7e, f). These results demonstrated that transcriptional regulation of HIF-1α and its target gene such as ID1 were important in human leukemia cells which might be deregulated by either *ASXL1* or *RUNX1* mutation or coexisted mutation of both genes (Fig. 8).

## Discussion

The prognosis of CMML is poor, and effective therapies are limited; CMML remains to be a challenging hematological malignancy concerning the pathogenesis and treatment [33]. With the introduction of next-generation sequencing technology in the past decade, it is well known that CMML is characterized by the presence of various somatic mutations of driver genes [6, 7, 34]. *ASXL1* mutations were common in CMML and frequently associated with a combination of various gene mutations [4, 34, 35]. We had analyzed the mutational status of various mutations related to myeloid neoplasms in a cohort of 110 patients with CMML. *ASXL1* mutations were present in 29 (26.4%) of CMML patients. *RUNX1* mutations were detected in 34 (30.9%) patients ([16] and updated). Among them, both *ASXL1* and *RUNX1* were mutated in 10% (11/110) of our patients. Moreover, 7 of the CMML patients carrying *ASXL1* mutations had sAML transformation later, 4 of them coexisted with *RUNX1* mutations at initial diagnosis of CMML, and additional 2 patients acquired *RUNX1* mutations at sAML transformation [36]. We had observed that 8 of 11 patients harbored *RUNX1* mutations located in the runt homology domain in *ASXL1*-mutated CMML [17]. On the other hand, we could not detect *ASXL1* or *RUNX1* mutations in CML patients at initial diagnosis of chronic phase; however, acquisition of *RUNX1* and/or *ASXL1* mutations occurred in 25.5% (13/51) of patients during AML transformation. Moreover, 3 of the 6 CML-myeloid BC patients carrying *ASXL1* mutations had co-existence of *RUNX1* and *ASXL1* mutations [18]. To further investigate the role of RUNX1 mutations to the myeloid transformation in ASXL1-mutated leukemia, we performed in vitro and in vivo expressing either *ASXL1* or *RUNX1* single mutant or combination of *ASXL1* with *RUNX1* mutant. In accordance with the augmentation of cell growth and impairment of differentiation of 32D cells, a murine myeloid cell line, self-renewal activity of primary murine BM cells, was also increased in the presence of the co-expression of ASXL1-R693X and RUNX1-R135T. Myeloid progenitor 32D-expressing *RUNX1* mutants blocked granulocytic differentiation by G-CSF [20]. It was also reported that either ASXL1-MT alone or the cooperation of SETBP1-D868N and ASXL1-MT impaired G-CSF-induced differentiation of 32D cells [14]. Recently, Nagase et al. reported that the expression of mutant Asxl1 resulted in the dysfunction of hematopoietic stem and progenitor cells, perturbed erythroid-lineage differentiation, and promoted leukemia transformation in vivo using conditional knock-in mouse [37]. Previously, Goldfarb found that loss-of-function or haploinsufficiency of *RUNX1* gene impaired megakaryopoiesis [38]. We also observed that the overexpression of *RUNX1*-R135T in K562 cells, a heterozygous *ASXL1* mutant CML cell line, augmented cell growth and impaired PMA-induced megakaryocytic differentiation as evidenced by the reduction of CD61 expression with immature cell morphology in *RUNX1*-R135T-expressing K562 cells. In contrast, either BCR-ABL1 or ABL1 expressions were not changed in R135T-expressing K562 cells. The K562 cell line is a multipotent leukemia cell line that undergoes megakaryocytic differentiation upon PMA induction with enhanced expression of surface antigens such as CD41 and CD61 [26]. We also found that overexpression of *RUNX1*-R135T in *ASXL1* mutant K562 cells upregulated *HOXA* genes; however, the expression of H3K27me3 was not changed in mutant cells.

It has been demonstrated that overexpression of *ID1* immortalized myeloid progenitors in vitro and led to MPD in vivo [39]. Moreover, high *ID1* expression was associated with poor outcome in AML with shorter event-free and overall survival [40]. We found that *ID1* mRNA and protein levels were elevated in the BM, spleen, and liver samples of mice carrying both *ASXL1*-R693X and *RUNX1*-R135T mutants compared to either *ASXL1*-R693X or *RUNX1*-R135T mutant and control mice. Similarly, *ID1* expression in BM cells from CMML patients carrying both *ASXL1* and *RUNX1* mutations was not only higher than normal BM cells but also much higher than either *ASXL1*-MT or *RUNX1*-MT alone. ID1 plays a critical role in the leukemogenesis of AML through regulation and interaction with AKT1 [41]. The activation of AKT signaling is an important mechanism of transformation to AML, and the effects of ID1 on leukemogenesis through AKT has been reported [41, 42]. In line with this, we observed the upregulation of AKT1 signaling with the enhancement of ID1 expression in coexisted *ASXL1* and *RUNX1* mutant cells that would contribute to the leukemogenesis in a subset of patients with CMML or CML myeloid BC (Fig. 8).

RUNX1 and HIF-1α directly interacted with each other, in which the runt homology domain of RUNX1 was mainly involved [43]. Overexpression of RUNX1-WT inhibited DNA binding and transcriptional activity of HIF-1 protein with reduced expression of HIF-1-targeted genes [43]. HIF-1α is a master transcriptional regulator that maintains HSC cell cycle regulation and activates the transcription of genes that are involved in critical aspects of cancer biology, including angiogenesis, cell survival, and invasion [43–45]. We observed that both RUNX1-WT and RUNX1-R135T could interact with HIF-1α; however, RUNX-R135T might have more interaction with HIF-1α. Moreover, we showed that overexpression of RUNX1-R135T or combined expression of ASXL1-R693X and RUNX1-R135T enhanced transcriptional activity of HIF-1α and its target gene, *ID1* expression. One possible explanation of this finding was that more stable RUNX1-R135T protein might compete with RUNX1-WT protein for DNA binding and β-heterodimerization and reduced RUNX1-mediated transcriptional activity in the cells. Hence, mutant RUNX1-R135T increased HIF1-α activity and its target gene expression. Previously, we systematically analyzed the biologic activities of RUNX1 mutants identified from patients with CMML and MDS by in vitro functional assays [19]. We observed that most RUNX1 mutants had reduced abilities in DNA binding, CBF-β heterodimerization, and C-FMS gene induction, especially missense mutations at runt homology domain, but we did not use RUNX1-R135T mutant for these functional experiments in that study. HIF-1α is a well-established transcriptional regulator of VEGF. Other investigators demonstrated that Id-1 induced angiogenesis through HIF-1α-mediated VEGF activation in human endothelial cells, breast cancer, and hepatocellular carcinoma [46–48]. Our results of reduction of ID1 expression by the inhibition of HIF-1α in transformed leukemia cells supports that ID1 is controlled by HIF-1α, which might be deregulated by either *ASXL1* or *RUNX1* mutation or coexisted mutant of both genes. Our ChIP data following co-expression of *ASXL1* and *RUNX1* mutations recognized the deregulation of *ID1* in myeloid leukemia cells, suggesting the role of myeloid leukemia transformation by combined mutations were at least partly attributed to the upregulated *ID1* expression (Fig. 8). Data presented here showed that either *ASXL1*-R693X or *RUNX1*-R135T mutant alone did not have much effect on leukemia cells; however, cooperative mutations led to the enhancement of HIF-1α recruitments to the promoter region of the *ID1* gene. This suggested that the increase was not only due to the upregulation and stabilization of HIF-1α by the RUNX1 mutant, but also ASXL1 plays a significant role in this finding. Both ASXL1 and ID1 were physically interacted with AKT1 [41, 49], and ASXL1/AKT1/ID1 axis may regulate HIF-1α expression in combined ASXL1 and RUNX1-mutated cells of which underlying mechanism remains to be explored. Notably, the leukemic cells from our patients harboring coexisted mutations of *ASXL1* and *RUNX1* correlated with the upregulation of *ID1* gene expression, supporting the role of cooperative mutation of *ASXL1* and *RUNX1* on ID1 expression and myeloid leukemia transformation.

## Conclusions

Taken together, we found that the gain-of-function of RUNX1-MT enhanced among *ASXL1*-mutated leukemia in vitro and in vivo. Our results demonstrated that RUNX1-MT have critical roles in the leukemia transformation including augmentation of cell proliferation, blocked differentiation, and increased self-renewal activity in *ASXL1*-mutated cells. RUNX1 mutant directly enhanced the transcriptional activity of HIF-1α and increased its target gene expression such as *ID1* which could be a potential target for future therapy in *ASXL1*-mutated leukemia.

## Supplementary information

**Additional file 1: Table S1.** List of qRT-PCR primer sets to check mRNA expression of different genes. **Figure S1.** Simultaneous expression of ASXL1-R693X and RUNX1-R135T augmented cell proliferation. **Figure S2.** Co-expression of ASXL1-R693X and RUNX1-R135T in U937 cells and transformed cells had no effect on CD11b expression in the presence of DMSO. **Figure S3.** *ASXL1*-mutant K562 cell line expressed H3K27me3 and endogenous ASXL1 in K562 cells interacted with endogenous EZH2 protein, and K562 transformed cells had no effect on CD61 expression in the presence of DMSO. **Figure S4.** ASXL1-R693X collaborates with RUNX1-R135T in myeloid transformation. **Figure S5.** Collaborative mutations of ASXL1-R693X and RUNX1-R135T enhanced ID1 expression. **Figure S6.** RUNX1 and HIF-1α do not interacted with ASXL1. **Figure S7.** Stability of RUNX1-R135T mutant protein is more than RUNX1-WT protein. **Figure S8.** Collaboration of RUNX1-R135T with ASXL1-R693X in the augmentation of HIF-1α and its target gene.

**Additional file 2: Dataset S1.** Gene expression microarray analysis of RUNX1-WT, RUNX1-R135T mutant and EV control stably expressed in K562 cell line. List of upregulated genes (> 2 fold) in RUNX1-R135T cells compared to EV.

## Data Availability

All data generated or analyzed during this study are included in this published article and its supplementary information files. Raw and processed data are available upon request.

## Abbreviations

AML: Acute myeloid leukemia
ASXL1: Additional sex comb-like 1
BC: Blast crisis
BM: Bone marrow
BMC: Bone marrow cells
BMT: Bone marrow transplantation
CFU: Colony-forming unit
ChIP: Chromatin immunoprecipitation
CML: Chronic myeloid leukemia
CMML: Chronic myelomonocytic leukemia
CoCl_2_: Cobalt chloride
DMSO: Dimethyl sulfoxide
EV: Empty vector
G-CSF: Granulocyte colony-stimulating factor
H3K27me3: Histone-3-lysine-27 trimethylation
H3K4me3: Histone-3-lysine-4 trimethylation
HIF-1α: Hypoxia-inducible factor-1α
HRE: Hypoxia-responsive element
ID1: Inhibitor of DNA binding 1
MDS: Myelodysplastic syndromes
MT: Mutant
PCR: Polymerase chain reaction
PMA: Phorbol 12-myristate 13-acetate
RT-qPCR: Real-time quantitative PCR
RUNX1: Runt-related transcription factor 1
sAML: Secondary AML
SDS–PAGE: Sodium dodecyl sulfate polyacrylamide gel electrophoresis
WB: Western blot
WT: Wild-type

## References

[1] Gelsi-Boyer V, Brecqueville M, Devillier R, Murati A, Mozziconacci MJ, Birnbaum D (2012). Mutations in ASXL1 are associated with poor prognosis across the spectrum of malignant myeloid diseases. J Hematol Oncol.

[2] Abdel-Wahab O, Adli M, LaFave LM, Gao J, Hricik T, Shih AH, Pandey S, Patel JP, Chung YR, Koche R (2012). ASXL1 mutations promote myeloid transformation through loss of PRC2-mediated gene repression. Cancer Cell.

[3] Vannucchi AM, Biamonte F (2011). Epigenetics and mutations in chronic myeloproliferative neoplasms. Haematologica.

[4] Gelsi-Boyer V, Trouplin V, Roquain J, Adélaïde J, Carbuccia N, Esterni B, Finetti P, Murati A, Arnoulet C, Zerazhi H (2010). ASXL1 mutations is associated with poor prognosis and acute transformation in chronic myelomonocytic leukemia. Br J Haematol.

[5] Boultwood J, Perry J, Zaman R, Fernandez-Santamaria C, Littlewood T, Kusec R, Pellagatti A, Wang L, Clark RE, Wainscoat JS (2010). High-density single nucleotide polymorphism array analysis and ASXL1 gene mutation screening in chronic myeloid leukemia during disease progression. Leukemia.

[6] Jankowska AM, Makishima H, Tiu RV, Szpurka H, Huang Y, Traina F, Visconte V, Sugimoto Y, Prince C, O'Keefe C (2011). Mutational spectrum analysis of chronic myelomonocytic leukemia includes genes associated with epigenetic regulation: UTX, EZH2, and DNMT3A. Blood.

[7] Arber DA, Orazi A, Hasserjian R, Thiele J, Borowitz MJ, Le Beau MM, Bloomfield CD, Cazzola M, Vardiman JW (2016). The 2016 revision to the World Health Organization classification of myeloid neoplasms and acute leukemia. Blood.

[8] Patnaik MM, Wassie EA, Lasho TL, Hanson CA, Ketterling R, Tefferi A (2015). Blast transformation in chronic myelomonocytic leukemia: risk factors, genetic features, survival, and treatment outcome. Am J Hematol.

[9] Wassie EA, Itzykson R, Lasho TL, Kosmider O, Finke CM, Hanson CA, Ketterling RP, Solary E, Tefferi A, Patnaik MM (2014). Molecular and prognostic correlates of cytogenetic abnormalities in chronic myelomonocytic leukemia: a Mayo Clinic-French Consortium Study. Am J Hematol.

[10] Pratcorona M, Abbas S, Sanders MA, Koenders JE, Kavelaars FG, Erpelinck-Verschueren CA, Zeilemakers A, Lowenberg B, Valk PJ (2012). Acquired mutations in ASXL1 in acute myeloid leukemia: prevalence and prognostic value. Haematologica.

[11] Lindsley RC, Mar BG, Mazzola E, Grauman PV, Shareef S, Allen SL, Pigneux A, Wetzler M, Stuart RK, Erba HP (2015). Acute myeloid leukemia ontogeny is defined by distinct somatic mutations. Blood.

[12] Asada S, Goyama S, Inoue D, Shikata S, Takeda R, Fukushima T, Yonezawa T, Fujino T, Hayashi Y, Kawabata KC (2018). Mutant ASXL1 cooperates with BAP1 to promote myeloid leukaemogenesis. Nat Commun.

[13] Uni M, Masamoto Y, Sato T, Kamikubo Y, Arai S, Hara E, Kurokawa M (2018). Modeling ASXL1 mutation revealed impaired hematopoiesis caused by derepression of p16Ink4a through aberrant PRC1-mediated histone modification. Leukemia.

[14] Inoue D, Kitaura J, Matsui H, Hou HA, Chou WC, Nagamachi A, Kawabata KC, Togami K, Nagase R, Horikawa S (2015). SETBP1 mutations drive leukemic transformation in ASXL1-mutated MDS. Leukemia.

[15] Yang H, Kurtenbach S, Guo Y, Lohse I, Durante MA, Li J, Li Z, Al-Ali H, Li L, Chen Z (2018). Gain of function of ASXL1 truncating protein in the pathogenesis of myeloid malignancies. Blood.

[16] Kuo MC, Liang DC, Huang CF, Shih YS, Wu JH, Lin TL, Shih LY (2009). RUNX1 mutations are frequent in chronic myelomonocytic leukemia and mutations at the C-terminal region might predict acute myeloid leukemia transformation. Leukemia.

[17] Kuo MC, Shih LY, Tsai SC, Liang ST, Huang YJ, Shih YS, Lin TH, Lai CY, Liang DC (2015). RUNX1 mutation and low RUNX1 transactivating activity predict higher risk of AML transformation and inferior leukemia-free survival in chronic myelomonocytic leukemia. Haematologica.

[18] Kao HW, Kuo MC, Wang PN, Dunn P, Wu JH, Lai CL, Lin TH, Shih LY (2016). Evolution of RUNX1 and ASXL1 mutations during progression of chronic myeloid leukemia to myeloid blast phase: an analysis of 52 matched paired samples at both. Haematologica.

[19] Tsai SC, Shih LY, Liang ST, Huang YJ, Kuo MC, Huang CF, Shih YS, Lin TH, Chiu MC, Liang DC (2015). Biological activities of RUNX1 mutants predict secondary acute leukemia transformation from chronic myelomonocytic leukemia and myelodysplastic syndromes. Clin Cancer Res.

[20] Zhao LJ, Wang YY, Li G, Ma LY, Xiong SM, Weng XQ, Zhang WN, Wu B, Chen Z, Chen SJ (2012). Functional features of RUNX1 mutants in acute transformation of chronic myeloid leukemia and their contribution to inducing murine full-blown leukemia. Blood.

[21] Liang DC, Liu HC, Yang CP, Jaing TH, Hung IJ, Yeh TC, Chen SH, Hou JY, Huang YJ, Shih YS (2013). Cooperating gene mutations in childhood acute myeloid leukemia with special reference on mutations of ASXL1, TET2, IDH1, IDH2, and DNMT3A. Blood.

[22] Bera R, Chiu MC, Huang YJ, Liang DC, Lee YS, Shih LY (2018). Genetic and epigenetic perturbations by DNMT3A-R882 mutants impaired apoptosis through augmentation of PRDX2 in myeloid leukemia cells. Neoplasia.

[23] Bersenev A, Wu C, Balcerek J, Jing J, Kundu M, Blobel GA, Chikwava KR, Tong W (2010). Lnk constrains myeloproliferative diseases in mice. J Clin Invest.

[24] Robert-Richard E, Ged C, Ortet J, Santarelli X, Lamrissi-Garcia I, de Verneuil H, Mazurier F (2006). Human cell engraftment after busulfan or irradiation conditioning of NOD/SCID mice. Haematologica.

[25] Wilkinson FL, Sergijenko A, Langford-Smith KJ, Malinowska M, Wynn RF, Bigger BW (2013). Busulfan conditioning enhances engraftment of hematopoietic donor-derived cells in the brain compared with irradiation. Mol Ther.

[26] Navarro F, Gutman D, Meire E, Caceres M, Rigoutsos I, Bentwich Z, Lieberman J (2009). miR-34a contributes to megakaryocytic differentiation of K562 cells independently of p53. Blood.

[27] Jacquel A, Herrant M, Defamie V, Belhacene N, Colosetti P, Marchetti S, Legros L, Deckert M, Mari B, Cassuto JP (2006). A survey of the signaling pathways involved in megakaryocytic differentiation of the human K562 leukemia cell line by molecular and c-DNA array analysis. Oncogene.

[28] Bera R, Liang DC, Chiu MC, Huang YJ, Liang ST, Shih LY (2012). PHD domain deletion mutations of ASXL1 promote myeloid leukemia transformation through epigenetic dysregulation and inhibit megakaryocytic differentiation through the inactivation of FOSB in K562 cells. Blood.

[29] Jankovic V, Ciarrocchi A, Boccuni P, DeBlasio T, Benezra R, Nimer SD (2007). Id1 restrains myeloid commitment, maintaining the self-renewal capacity of hematopoietic stem cells. Proc Natl Acad Sci U S A.

[30] Mistry H, Hsieh G, Buhrlage SJ, Huang M, Park E, Cuny GD, Galinsky I, Stone RM, Gray NS, D’Andrea AD, Parmar K (2013). Small-molecule inhibitors of USP1 target ID1 degradation in leukemic cells. Mol Cancer Ther.

[31] Peng ZG, Zhou MY, Huang Y, Qiu JH, Wang LS, Liao SH, Dong S, Chen GQ (2008). Physical and functional interaction of Runt-related protein 1 with hypoxia-inducible factor-1alpha. Oncogene.

[32] Fu B, Xue J, Li Z, Shi X, Jiang BH, Fang J (2007). Chrysin inhibits expression of hypoxia-inducible factor-1alpha through reducing hypoxia-inducible factor-1alpha stability and inhibiting its protein synthesis. Mol Cancer Ther.

[33] Patnaik MM, Tefferi A (2016). Chronic myelomonocytic leukemia: 2016 update on diagnosis, risk stratification, and management. Am J Hematol.

[34] Patnaik MM, Parikh SA, Hanson CA, Tefferi A (2014). Chronic myelomonocytic leukaemia: a concise clinical and pathophysiological review. Br J Haematol.

[35] Carbuccia N, Trouplin V, Gelsi-Boyer V, Murati A, Rocquain J, Adelaide J, Olschwang S, Xerri L, Vey N, Chaffanet M (2010). Mutual exclusion of ASXL1 and NPM1 mutations in a series of acute myeloid leukemias. Leukemia.

[36] Su YJ, Kuo MC, Wang PN, Wu JH, Lin YH, Huang TY, Lin TL, Chang H, Hung YS, Shih LY. Genetic evolution patterns in patients with chronic myelomonocytic leukemia to secondary acute myeloid leukemia: an analysis of 36 paired samples. The 24th Congress of the European Hematology Association, Amsterdam, The Netherlands, June 13–16, 2019.

[37] Nagase R, Inoue D, Pastore A, Fujino T, Hou HA, Yamasaki N, Goyama S, Saika M, Kanai A, Sera Y (2018). Expression of mutant Asxl1 perturbs hematopoiesis and promotes susceptibility to leukemic transformation. J Exp Med.

[38] Goldfarb AN (2009). Megakaryocytic programming by a transcriptional regulatory loop: a circle connecting RUNX1, GATA-1, and P-TEFb. J Cell Biochem.

[39] Suh HC, Leeanansaksiri W, Ji M, Klarmann KD, Renn K, Gooya J, Smith D, McNiece I, Lugthart S, Valk PJ (2008). Id1 immortalizes hematopoietic progenitors in vitro and promotes a myeloproliferative disease in vivo. Oncogene.

[40] Tang R, Hirsch P, Fava F, Lapusan S, Marzac C, Teyssandier I, Pardo J, Marie JP, Legrand O (2009). High Id1 expression is associated with poor prognosis in 237 patients with acute myeloid leukemia. Blood.

[41] Wang L, Man N, Sun XJ, Tan Y, Garcia-Cao M, Liu F, Hatlen M, Xu H, Huang G, Mattlin M (2015). Regulation of AKT signaling by Id1 controls t (8;21) leukemia initiation and progression. Blood.

[42] Kharas MG, Okabe R, Ganis JJ, Gozo M, Khandan T, Paktinat M, Gilliland DG, Gritsman K (2010). Constitutively active AKT depletes hematopoietic stem cells and induces leukemia in mice. Blood.

[43] Takubo K, Goda N, Yamada W, Iriuchishima H, Ikeda E, Kubota Y, Shima H, Johnson RS, Hirao A, Suematsu M, Suda T (2010). Regulation of the HIF-1α level is essential for hematopoietic stem cells. Cell Stem Cell.

[44] Semenza GL (2003). Targeting HIF-1 for cancer therapy. Nat Rev Cancer.

[45] Kim HJ, Chung H, Yoo YG, Kim H, Lee JY, Lee MO, Kong G (2007). Inhibitor of DNA binding 1 activates vascular endothelial growth factor through enhancing the stability and activity of hypoxia-inducible factor-1alpha. Mol Cancer Res.

[46] Shweiki D, Itin A, Soffer D, Keshet E (1992). Vascular endothelial growth factor induced by hypoxia may mediate hypoxia-initiated angiogenesis. Nature.

[47] Forsythe JA, Jiang BH, Iyer NV, Agani F, Leung SW, Koos RD, Semenza GL (1996). Activation of vascular endothelial growth factor gene transcription by hypoxia-inducible factor 1. Mol Cell Biol.

[48] Lee TK, Poon RT, Yuen AP, Ling MT, Wang XH, Wong YC, Guan XY, Man K, Tang ZY, Fan ST (2006). Regulation of angiogenesis by Id-1 through hypoxia-inducible factor-1alpha-mediated vascular endothelial growth factor up-regulation in hepatocellular carcinoma. Clin Cancer Res.

[49] Youn HS, Kim TY, Park UH, Moon ST, An SJ, Lee YK, Hwang JT, Kim EJ, Um SJ (2017). Asxl1 deficiency in embryonic fibroblasts leads to cellular senescence via impairment of the AKT-E2F pathway and Ezh2 inactivation. Sci Rep.

