# Supplementary material for: RUNX1 mutations promote leukemogenesis of myeloid malignancies in ASXL1-mutated leukemia
Source: J Hematol Oncol. 2019 Oct 22;12:104. doi: 10.1186/s13045-019-0789-3 (PMC6805634; doi:10.1186/s13045-019-0789-3)
Supplement: Supplementary file 1 — Additional file 1: Table S1. List of qRT-PCR primer sets to check mRNA expression of different genes. Figure S1. Simultaneous expression of ASXL1-R693X and RUNX1-R135T augmented cell proliferation. Figure S2. Co-expression of ASXL1-R693X and RUNX1-R135T in U937 cells and transformed cells had no effect on CD11b expression in the presence of DMSO. Figure S3. ASXL1-mutant K562 cell line expressed H3K27me3 and endogenous ASXL1 in K562 cells interacted with endogenous EZH2 protein, and K562 transformed cells had no effect on CD61 expression in the presence of DMSO. Figure S4. ASXL1-R693X collaborates with RUNX1-R135T in myeloid transformation. Figure S5. Collaborative mutations of ASXL1-R693X and RUNX1-R135T enhanced ID1 expression. Figure S6. RUNX1 and HIF-1α do not interacted with ASXL1. Figure S7. Stability of RUNX1-R135T mutant protein is more than RUNX1-WT protein. Figure S8. Collaboration of RUNX1-R135T with ASXL1-R693X in the augmentation of HIF-1α and its target gene. [file 13045_2019_789_MOESM1_ESM.pdf]

Supplementary Information

Table S1. List of qRT-PCR primer sets to check mRNA expression of different genes

Gene	Forward	Reverse	Size (bp)
HOXA5	GGCTACAATGGCATGGATCT	CTGAGGAGAGTGCGTGGAC	150
HOXA7	ACTTCAACCGCTACCTGACG	CAGTCGGACCTTCGTCCTTA	138
HOXA9	CCACGCTTGACACTCACACT	CGCTCTCATTCTCAGCATTG	120
HOXA10	GGATTCCCTGGGCAATTCCA	CTAATCTCTAGGCGCCGCTC	162
ID1	ACGTGCTGCTCTACGACATGA	GAATCTCCACCTTGCTCACCTT	96
***Id1* (mouse)**	ACGAGCAGCAGGTGAACGT	TTGCTCACTTTGCGGTTCTG	100
ASXL1	AGT CCA CGG ATA CAG CCT CT	ACG CAT GTC ACC ATT CAC CT	164
RUNX1	GGCTGGCAATGATGAAAACTA	TTTTGATGGCTCTGTGGTAGG	185

Figure S1.

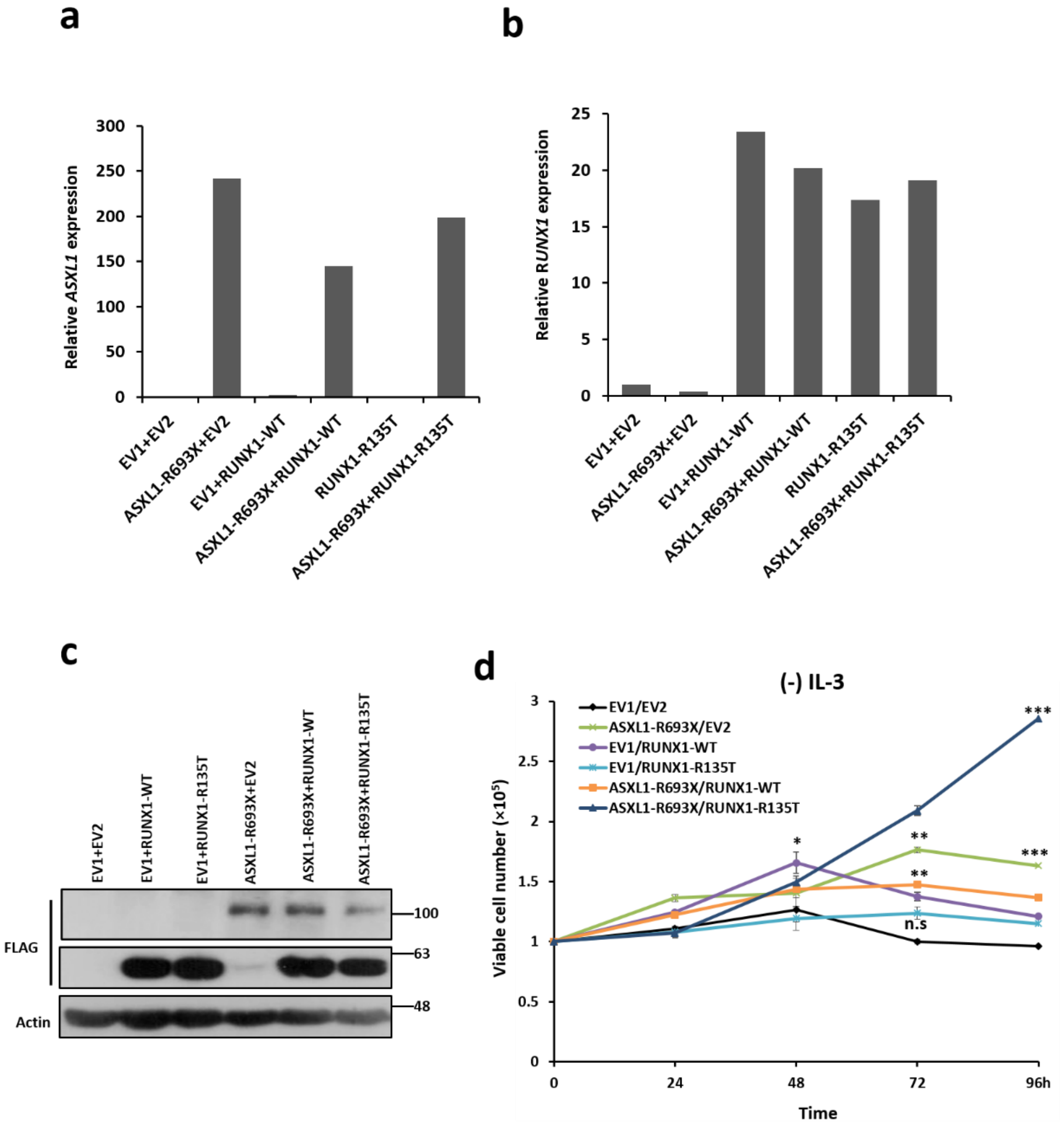

Fig. S1 Simultaneous expression of ASXL1-R693X and RUNX1-R135T augmented cell proliferation (related to Figure 1). **(a-b)** Relative mRNA levels expression of *ASXL1* (**a**) and *RUNX1* (**b**) WT/MT evaluated by qRT-PCR analyses of 32D cell lines overexpressed by different plasmids. (**c**) Stably-expressed FLAG-tagged RUNX1-WT/R135T and truncated ASXL1 protein R693X in 32D cells were detected by immunoblot analyses using FLAG antibody. (**d)** IL-3 dependent murine myeloid 32D cells stably transduced with ASXL1-R693X, RUNX1-WT/R135T and combination of ASXL1-R693X and RUNX1-R135T were cultured in the absence of IL-3. Number of viable cells were counted at different time points. All data error bars in represented here are the mean ±S.D. of duplicate cultures and repeated twice; **P*<0.05, ***P*<0.03, ****P*<0.01, n.s. not significant.

Figure S2.

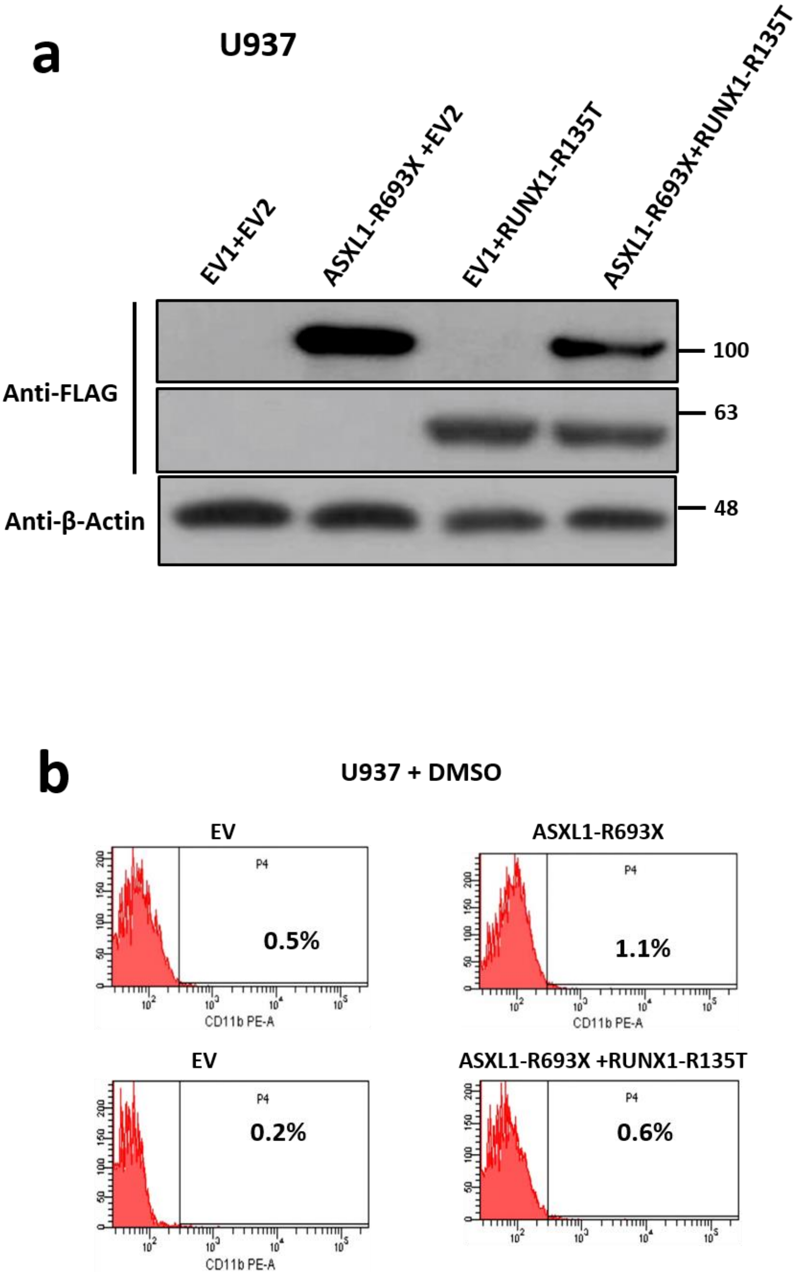

Fig. S2 Co-expression of ASXL1-R693X and RUNX1-R135T in U937 cells and transformed cells had no effect on CD11b expression in the presence of DMSO (related to Figure 1e). **(a)** ASXL1-R693X, RUNX1-R135T and combination of two mutants were stably expressed in U937 cells, and expression was checked by immunoblotting with FLAG-antibody. (**b**) Flow cytometry analyses of CD11b after the treatment of DMSO in transduced U937 cells for 96 h; a representative experiment is shown.

Figure S3.

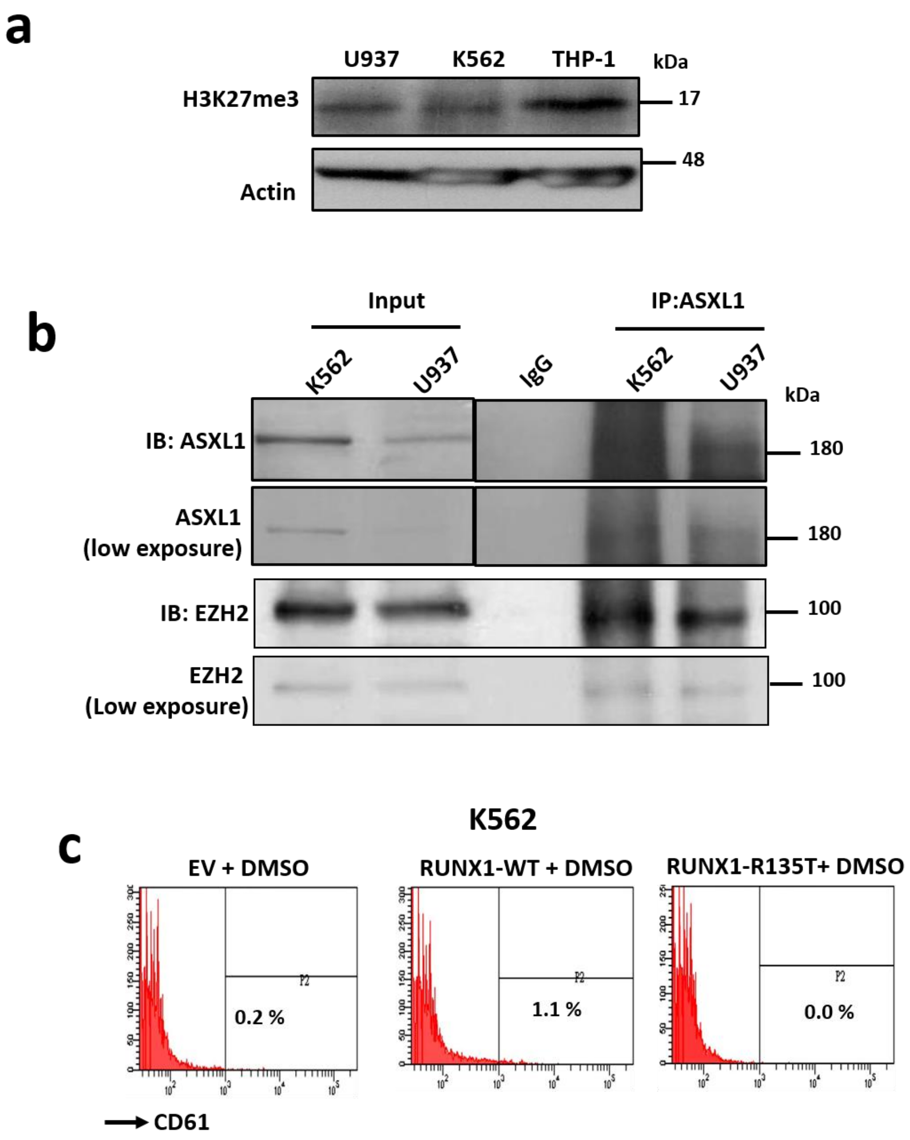

Fig. S3 *ASXL1*-mutant K562 cell line expressed H3K27me3 and endogenous ASXL1 interacted with endogenous EZH2 protein, and K562 transformed cells had no effect on CD61 expression in the presence of DMSO (related to Figure 2). (**a**) Immunoblot of endogenous expression of H3K27me$_3$ in different leukemia cell lines including *ASXL1*-mutant K562 cell line. (**b**) Endogenous ASXL1 in K562 and U937 cells were immunoprecipitated with ASXL1 primary antibody and immunoblotted with ASXL1 and EZH2 antibody. (**c**) Flow cytometry analyses of megakaryocytic marker (CD61) after the treatment of DMSO in transduced K562 cells for 96 h; a representative experiment is shown.

Figure S4.

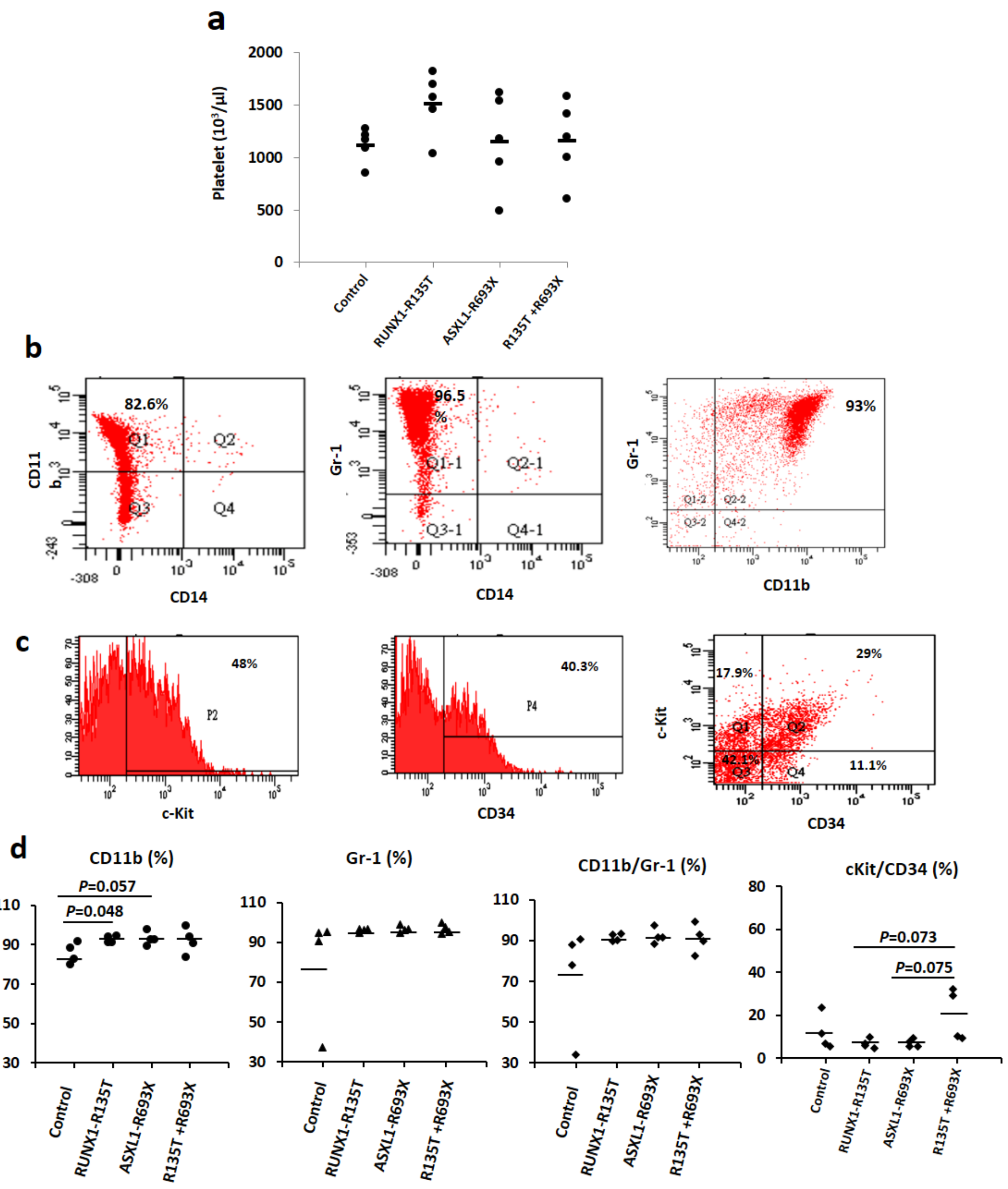

Fig. S4 ASXL1-R693X collaborates with RUNX1-R135T in myeloid transformation (related to Figure 4). **(a)** Platelets count in peripheral blood of transduced mice (n=5 for each group). **(b-c)** Flow cytometric analyses of BM cells derived from representative of disease mice with ASXL1-R693X and RUNX1-R135T are shown. (**d**) Flow cytometric analyses of BM cells derived from mice transplanted with ASXL1-R693X, RUNX1-R135T, combined expression of ASXL1-R693X and RUNX1-R135T, and EV control (n=4 of each group). *P*-value showing calculated as indicated in figures.

Figure S5

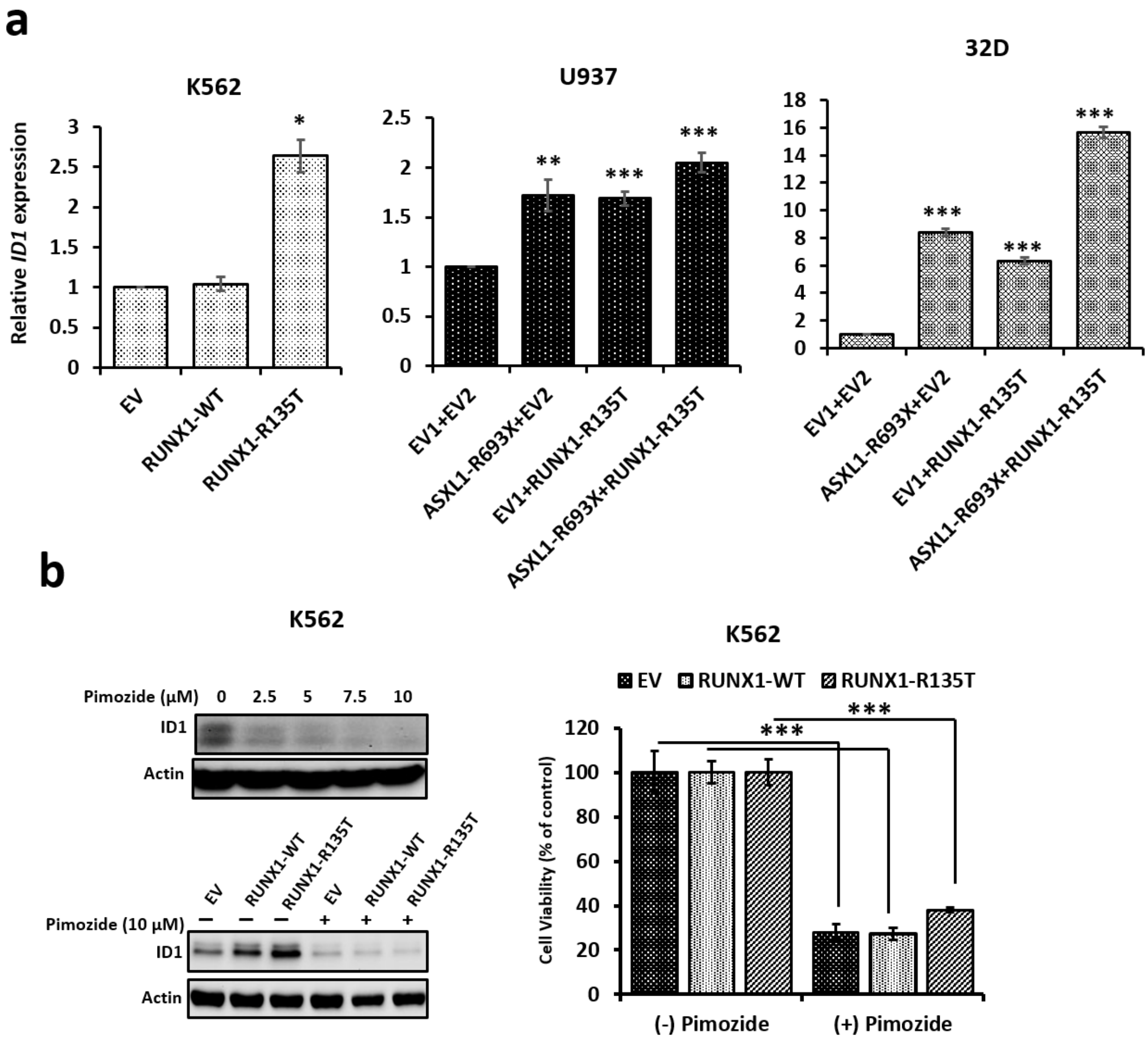

Fig. S5 Collaborative mutations of ASXL1 and RUNX1 enhanced ID1 expression (related to figure 5). (a) Analysis of mRNA expression of *ID1* by RT-QPCR after stably overexpression of WT and mutant RUNX1 in K562 cells; ASXL1-R693X, RUNX1-R135T, and coexisted mutations of ASXL1 and RUNX1 in U937 and 32D cells. Error bars represent the mean ±S.D. based on two independent experiments. (b) K562 cells were treated with different dose of pimozide (purchased from BioVision Inc. CA, USA) for 48 h. The cells were harvested, lysed and immunoblotted with indicated antibodies. Stably-transduced K562 cells were grown with DMSO or 10 µM pimozide in triplicate for 48 h and counted viable cells. Similar cells were used for immunoblot analysis to check the ID1 expression. Actin representing as a loading control. Data represented here are the mean ±S.D. of triplicate cultures and experiments repeated twice. *P<0.05, **P<0.03, ***P<0.01, compared with the control or as indicated in figures.

Figure S6

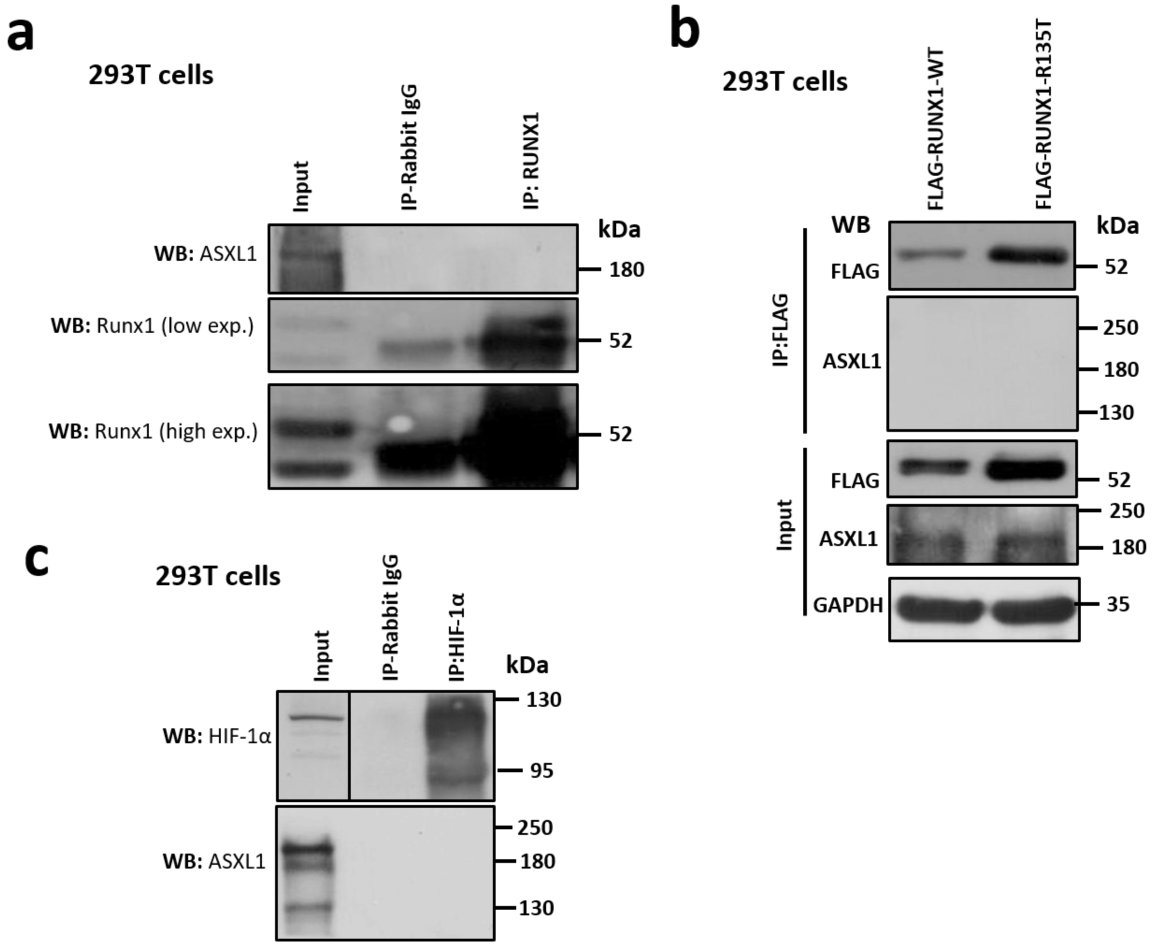

Fig. S6 RUNX1 and HIF-1α not interacted with ASXL1. a-b) Endogenous RUNX1 (a) or transiently-expressed FLAG-tagged RUNX1-WT/MT (b) in HEK-293T cells Immunoprecipitated with either RUNX1 antibody or FLAG antibody and immunoblot was performed using indicated antibodies. c) HEK-293T cells treated with 100 μM $CoCl_2$ for 24 h and Immunoprecipitation was performed using anti-HIF-1α antibody. Immunoblot was performed using ASXL1 and HIF-1α antibody. GAPDH representing as a loading control.

Figure S7

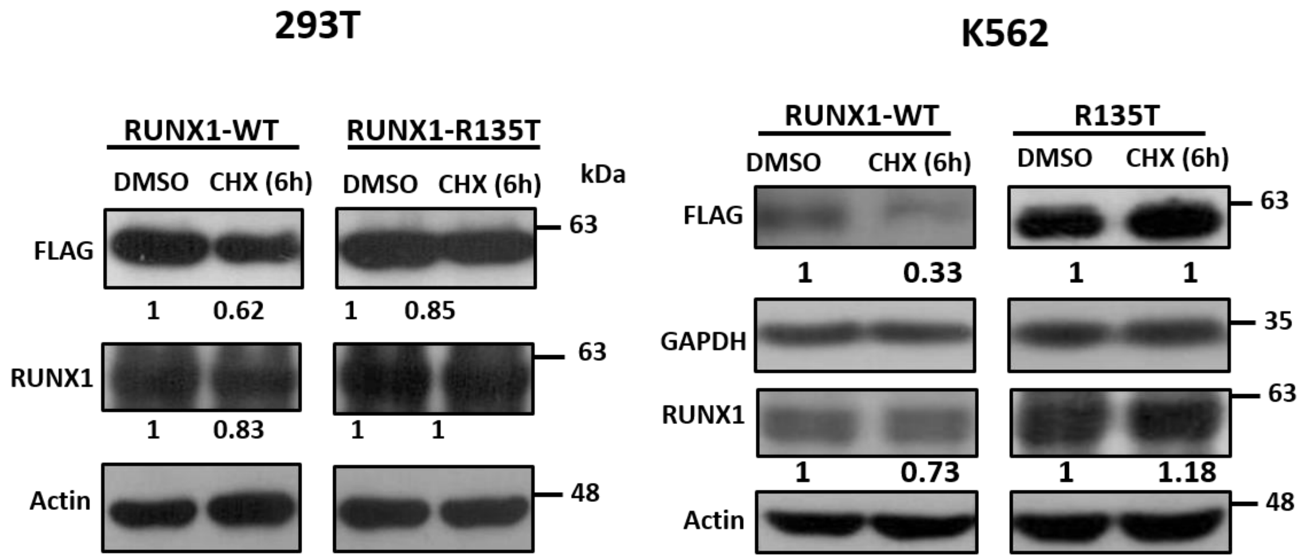

Fig. S7 Stability of RUNX1-R135T mutant protein is more than RUNX1-WT protein (relative to Figure 6). Either transient transfection of HEK293T cells with RUNX1-WT and RUNX1-R135T mutant or puromycin selected stable K562 cells followed by treatment with DMSO or cycloheximide (CHX) (100 μg/mL) for 6 h. Immunoblot results showed a larger time-dependent decrease in RUNX1-WT protein expression compared with RUNX1-R135T mutant protein. The values indicating relative density corresponding to Actin or GAPDH expression.

Figure S8

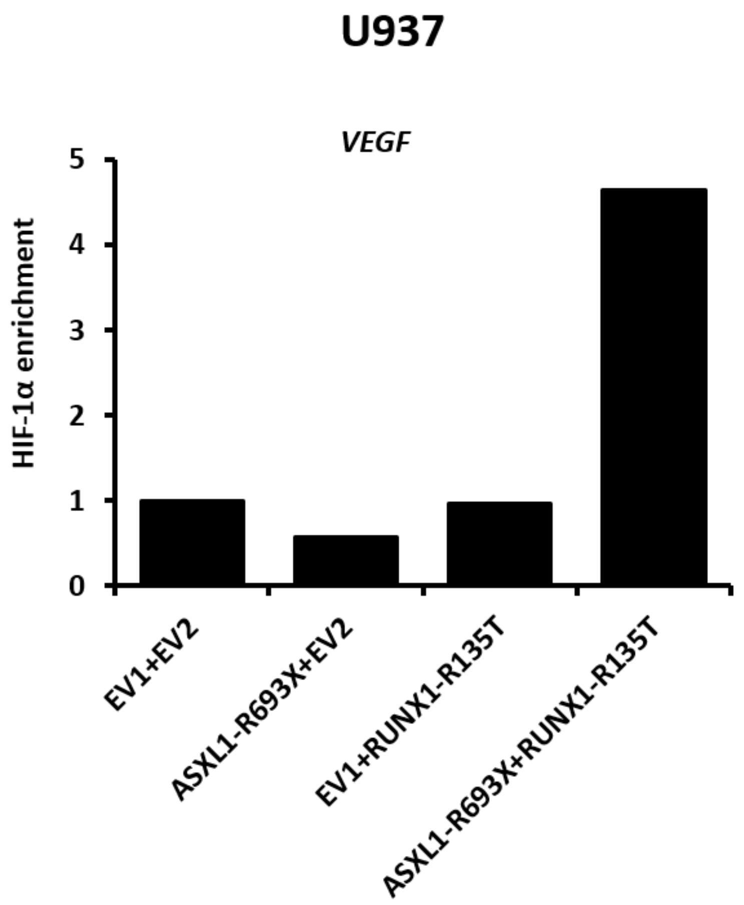

Fig. S8 Collaboration of RUNX1-R135T with ASXL1-R693X in the augmentation of HIF-1α and its target gene (related to Figure 7). Chromatin-immunoprecipitation (ChIP) analyses were performed using HIF1-α antibody in U937 cells overexpressed with *ASXL1*-R693X, *RUNX1*-R135T, *ASXL1*-R693X+*RUNX1*-R135T and *VEGF* expression was measured using real-time qPCR and ChIP-qPCR data is displayed as enrichment relative to input. All signals from transformed-U937 cells were standardized relative to signal from control U937 cells, which were set to 1.0.
