# Supplementary material for: RUNX1 mutations promote leukemogenesis of myeloid malignancies in ASXL1-mutated leukemia
Source: J Hematol Oncol. 2019 Oct 22;12:104. doi: 10.1186/s13045-019-0789-3 (PMC6805634; doi:10.1186/s13045-019-0789-3)
Supplement: Supplementary file 2 — Additional file 2: Dataset S1. Gene expression microarray analysis of RUNX1-WT, RUNX1-R135T mutant and EV control stably expressed in K562 cell line. List of upregulated genes (> 2 fold) in RUNX1-R135T cells compared to EV. [file 13045_2019_789_MOESM2_ESM.pdf]

Dataset S1:

Gene expression microarray analysis of RUNX1-WT, RUNX1-R135T mutant and EV control stably expressed in K562 cell line

List of Upregulated Genes >2 fold in RUNX1-R135T cells compared to EV

Gene Symbol	Gene Description	RUNX1-WT vs EV	RUNX1-R135T vs EV
HIPK2	homeodomain interacting protein kinase 2	0.37	13.12
SCG3	secretogranin III	0.78	10.42
LYVE1	lymphatic vessel endothelial hyaluronan receptor 1	0.72	8.02
ASXL1	additional sex combs like transcriptional regulator 1	1.05	6.90
ID1	inhibitor of DNA binding 1	0.58	4.30
MAGEC2	MAGE family member C2	1.02	4.14
IRS1	insulin receptor substrate 1	1.11	3.86
PDE9A	phosphodiesterase 9A	0.66	3.36
PLXDC2	plexin domain containing 2	0.64	3.21
SCRN1	secernin 1	1.01	3.58
ZBTB38	zinc finger and BTB domain containing 38	0.94	3.38
TRIM71	tripartite motif containing 71, E3 ubiquitin protein ligase	1.00	3.43
SH3BGRL3	SH3 domain binding glutamate-rich protein like 3	0.75	3.06
ASIC4	acid sensing ion channel family member 4	0.87	3.23
SLC19A1	solute carrier family 19 (folate transporter), member 1	1.13	3.14
MTND1P5	drially encoded NADH:ubiquinone oxidoreductase core subunit 1 pseu	1.16	3.09
MIR4503	microRNA 4503	1.00	3.17
AEBP1	AE binding protein 1	0.89	3.03
TLCD1	TLC domain containing 1	0.69	2.67
FRMD4A	FERM domain containing 4A	1.20	2.91
PLN	phospholamban	0.90	2.98
PLEK	pleckstrin	0.99	3.01
U1	U1 spliceosomal RNA	0.97	2.95
F2RL2	coagulation factor II (thrombin) receptor-like 2	0.80	2.65
RNU6-904P	RNA, U6 small nuclear 904, pseudogene	1.02	2.85
TMEM214	transmembrane protein 214	1.20	2.67
CD3EAP	CD3e molecule, epsilon associated protein	1.15	2.70
LOC100287392	Transcript Identified by AceView, Entrez Gene ID(s) 100287392	0.58	2.11
BCL9L	B-cell CLL/lymphoma 9-like	1.13	2.70
GRWD1	glutamate-rich WD repeat containing 1	0.83	2.62
FBXL14	F-box and leucine-rich repeat protein 14	0.94	2.75
FAM189B	family with sequence similarity 189, member B	0.87	2.67
RNU6-470P	RNA, U6 small nuclear 470, pseudogene	1.13	2.67
TOB2	transducer of ERBB2, 2	1.10	2.67
VAC14	Vac14 homolog (S. cerevisiae)	1.14	2.62
RPS23P5	somal protein S23 pseudogene 5 [Source:HGNC Symbol;Acc:HGNC:35	1.00	2.74
DHX34	DEAH (Asp-Glu-Ala-His) box polypeptide 34	1.13	2.61
MKL1	megakaryoblastic leukemia (translocation) 1	1.02	2.71
MIR1252	microRNA 1252	1.08	2.59
SPATA2	spermatogenesis associated 2	1.16	2.51

GNG12	guanine nucleotide binding protein (G protein), gamma 12	0.96	2.61
EHD1	EH domain containing 1	1.02	2.61
RITA1	RBPJ interacting and tubulin associated 1	1.19	2.43
H2AFX	H2A histone family, member X	0.90	2.50
ZFP36L2	ZFP36 ring finger protein-like 2	1.09	2.52
CAMK2B	Transcript Identified by AceView, Entrez Gene ID(s) 816	1.19	2.41
FABP5P1	fatty acid binding protein 5 pseudogene 1	1.19	2.41
CNOT3	CCR4-NOT transcription complex subunit 3	1.09	2.50
TTLL7	tubulin tyrosine ligase-like family member 7	0.82	2.34
WAS	Wiskott-Aldrich syndrome	0.98	2.54
MIR1261	microRNA 1261	1.12	2.41
SCOC-AS1	SCOC antisense RNA 1	1.14	2.39
ZFP36L1	ZFP36 ring finger protein-like 1	0.84	2.34
CAD	yl-phosphate synthetase 2, aspartate transcarbamylase, and dihydro	0.75	2.19
CD68	CD68 molecule	0.85	2.34
FARSA	phenylalanyl-tRNA synthetase alpha subunit	1.17	2.33
SLC25A44	solute carrier family 25, member 44	0.74	2.15
POM121C	POM121 transmembrane nucleoporin C	1.12	2.37
NTS	neurotensin	0.81	2.26
GMPPB	GDP-mannose pyrophosphorylase B	1.06	2.42
ZMIZ2	zinc finger, MIZ-type containing 2	0.76	2.16
MTSS1L	metastasis suppressor 1-like	1.20	2.28
AKT1S1	AKT1 substrate 1 (proline rich)	1.19	2.28
LGALS1	lectin, galactoside-binding, soluble, 1	1.15	2.31
RNU6-1333P	RNA, U6 small nuclear 1333, pseudogene	1.00	2.45
ATP13A1	ATPase type 13A1	0.90	2.34
Evf1_2	Embryonic ventral forebrain RNA 1 conserved region 2	1.00	2.44
SELT	selenoprotein T	1.05	2.38
LY6E	lymphocyte antigen 6 complex, locus E	0.81	2.18
DHCR24	24-dehydrocholesterol reductase	1.13	2.28
TAAR7P	trace amine associated receptor 7, pseudogene	0.82	2.19
FOXJ2	forkhead box J2	1.04	2.37
RNY4P13	RNA, Ro-associated Y4 pseudogene 13	1.01	2.39
ART2P	-ribosyltransferase 2, pseudogene [Source:HGNC Symbol;Acc:HGNC:7	0.97	2.35
TRIAP1	TP53 regulated inhibitor of apoptosis 1	1.19	2.19
TMUB1	transmembrane and ubiquitin-like domain containing 1	1.09	2.30
FUBP3	Transcript Identified by AceView, Entrez Gene ID(s) 8939	1.17	2.21
NDOR1	NADPH dependent diflavin oxidoreductase 1	0.90	2.27
STEAP3	STEAP family member 3, metalloreductase	0.75	2.02
TACC1	transforming, acidic coiled-coil containing protein 1	0.89	2.23
FBRS	fibrosin	0.77	2.04
MFSD12	major facilitator superfamily domain containing 12	1.17	2.17
EPN2	epsin 2	1.19	2.14
TEX261	testis expressed 261	1.18	2.14
CDK2AP2	cyclin-dependent kinase 2 associated protein 2	0.89	2.18
SEPN1	selenoprotein N, 1	0.97	2.26

SLC9A1	carrier family 9, subfamily A (NHE1, cation proton antiporter 1), mem	1.00	2.29
TRAPPC4	trafficking protein particle complex 4	1.19	2.09
TMEM184B	transmembrane protein 184B	1.15	2.13
KCNK5	potassium channel, two pore domain subfamily K, member 5	0.97	2.25
MAB21L2	mab-21-like 2 (C. elegans)	1.14	2.13
TIMM8A	translocase of inner mitochondrial membrane 8 homolog A (yeast)	1.00	2.27
TMEM109	transmembrane protein 109	1.12	2.14
ARHGDIA	Rho GDP dissociation inhibitor (GDI) alpha	1.07	2.18
EIF1AD	eukaryotic translation initiation factor 1A domain containing	1.16	2.09
CD53	CD53 molecule	1.01	2.24
SCARNA3	small Cajal body-specific RNA 3	1.15	2.10
ARHGAP22	Rho GTPase activating protein 22	1.13	2.12
SLC2A13P1	SLC2A13 pseudogene 1	1.00	2.24
RBM33	RNA binding motif protein 33	0.92	2.15
ISG20L2	interferon stimulated exonuclease gene 20kDa like 2	0.84	2.05
RAB35	RAB35, member RAS oncogene family	1.06	2.18
GYLTL1B	glycosyltransferase-like 1B	1.04	2.19
FYB	FYN binding protein	1.00	2.23
PRDX2P2	peroxiredoxin 2 pseudogene 2	0.95	2.17
PHLDA1	pleckstrin homology-like domain, family A, member 1	1.18	2.04
PTMS	parathymosin	1.02	2.19
MYRF	myelin regulatory factor	1.02	2.18
NRSN2	neurensin 2	1.09	2.11
PKN3	protein kinase N3	1.12	2.08
SURF2	surfeit 2 [Source:HGNC Symbol;Acc:HGNC:11475]	1.00	2.20
BICD2	bicaudal D homolog 2 (Drosophila)	0.97	2.17
PTRHD1	peptidyl-tRNA hydrolase domain containing 1	1.05	2.13
RBM26	ıczak2013 ANTISENSE, CDS, coding, INTERNAL best transcript NM_022	0.87	2.04
MIR1284	microRNA 1284	1.00	2.18
ATN1	atrophin 1	1.01	2.16
TRMU	tRNA 5-methylaminomethyl-2-thiouridylate methyltransferase	1.13	2.04
CREB3L2	cAMP responsive element binding protein 3-like 2	0.96	2.11
COX8A	cytochrome c oxidase subunit VIIIA (ubiquitous)	1.15	2.00
NOL6	nucleolar protein 6 (RNA-associated)	1.00	2.15
SLC9B1P1	3 (NHA1, cation proton antiporter 1), member 1 pseudogene 1 [Source	1.08	2.07
KDM4A-AS1	KDM4A antisense RNA 1	1.08	2.06
C17orf89	chromosome 17 open reading frame 89	1.13	2.01
NR1D1	nuclear receptor subfamily 1, group D, member 1	0.96	2.09
BOP1	block of proliferation 1	1.05	2.09
MATK	megakaryocyte-associated tyrosine kinase	0.90	2.02
EEF1A2	eukaryotic translation elongation factor 1 alpha 2	0.96	2.09
POLR2A	polymerase (RNA) II (DNA directed) polypeptide A, 220kDa	0.94	2.07
FJX1	four jointed box 1	1.08	2.04
TIMM8B	translocase of inner mitochondrial membrane 8 homolog B (yeast)	0.98	2.10
SEC61B	Sec61 translocon beta subunit	0.99	2.10
TMEM138	transmembrane protein 138	0.98	2.09

ND6	NADH dehydrogenase, subunit 6 (complex I)	0.95	2.05
OR9A2	olfactory receptor, family 9, subfamily A, member 2	1.00	2.10
DYNLT1	dynein, light chain, Tctex-type 1	0.93	2.02
SMCR8	Smith-Magenis syndrome chromosome region, candidate 8	1.08	2.01
NACC2	NACC family member 2, BEN and BTB (POZ) domain containing	0.97	2.06
HEPACAM2	HEPACAM family member 2	1.02	2.06
ZPLD1	zona pellucida-like domain containing 1	1.03	2.04
ABCB6	ATP binding cassette subfamily B member 6 (Langereis blood group)	1.02	2.05
FTSJ3	FtsJ homolog 3 (E. coli)	1.02	2.04
UBE2D3P4	ubiquitin conjugating enzyme E2D 3 pseudogene 4	1.00	2.04
NOL4L	nucleolar protein 4-like	1.01	2.01
FOXRED2	FAD-dependent oxidoreductase domain containing 2	1.01	2.01
DNAJA1P1	DnaJ heat shock protein family (Hsp40) member A1 pseudogene 1	-1.03	2.16
FOXM1	forkhead box M1	1.02	2.18
ASIC1	acid sensing ion channel 1	1.13	2.07
